# Jasmonate and ethylene dependent defence gene expression and suppression of fungal virulence factors: two essential mechanisms of Fusarium head blight resistance in wheat?

**DOI:** 10.1186/1471-2164-13-369

**Published:** 2012-08-02

**Authors:** Sven Gottwald, Birgit Samans, Stefanie Lück, Wolfgang Friedt

**Affiliations:** 1 Department of Plant Breeding, Justus-Liebig University, Institute of Agronomy and Plant Breeding I, Heinrich-Buff-Ring 26-32, Giessen, D-35392, Germany; 2 Biometry and Population Genetics, Justus-Liebig University, Institute of Agronomy and Plant Breeding II, Heinrich-Buff-Ring 26-32, Giessen, D-35392, Germany

## Abstract

**Background:**

Fusarium head blight (FHB) caused by *Fusarium* species like *F. graminearum* is a devastating disease of wheat (*Triticum aestivum*) worldwide. Mycotoxins such as deoxynivalenol produced by the fungus affect plant and animal health, and cause significant reductions of grain yield and quality. Resistant varieties are the only effective way to control this disease, but the molecular events leading to FHB resistance are still poorly understood. Transcriptional profiling was conducted for the winter wheat cultivars Dream (moderately resistant) and Lynx (susceptible). The gene expressions at 32 and 72 h after inoculation with *Fusarium* were used to trace possible defence mechanisms and associated genes. A comparative qPCR was carried out for selected genes to analyse the respective expression patterns in the resistant cultivars Dream and Sumai 3 (Chinese spring wheat).

**Results:**

Among 2,169 differentially expressed genes, two putative main defence mechanisms were found in the FHB-resistant Dream cultivar. Both are defined base on their specific mode of resistance. A non-specific mechanism was based on several defence genes probably induced by jasmonate and ethylene signalling, including lipid-transfer protein, thionin, defensin and GDSL-like lipase genes. Additionally, defence-related genes encoding jasmonate-regulated proteins were up-regulated in response to FHB. Another mechanism based on the targeted suppression of essential *Fusarium* virulence factors comprising proteases and mycotoxins was found to be an essential, induced defence of general relevance in wheat. Moreover, similar inductions upon fungal infection were frequently observed among FHB-responsive genes of both mechanisms in the cultivars Dream and Sumai 3.

**Conclusions:**

Especially ABC transporter, UDP-glucosyltransferase, protease and protease inhibitor genes associated with the defence mechanism against fungal virulence factors are apparently active in different resistant genetic backgrounds, according to reports on other wheat cultivars and barley. This was further supported in our qPCR experiments on seven genes originating from this mechanism which revealed similar activities in the resistant cultivars Dream and Sumai 3. Finally, the combination of early-stage and steady-state induction was associated with resistance, while transcript induction generally occurred later and temporarily in the susceptible cultivars. The respective mechanisms are attractive for advanced studies aiming at new resistance and toxin management strategies.

## Background

Fusarium head blight (FHB) caused e.g. by *F. graminearum* Schwabe (teleomorph *Gibberella zeae* (Schwein.) Petch) is one of the most destructive diseases of wheat (*T. aestivum* L.) worldwide, causing significant reductions in grain yield and quality. The most efficient strategy to control FHB in wheat is the use of resistant cultivars [1,2]. However, in hexaploid wheat the resistance to FHB is highly complex. Since 1999, over 200 QTL have been reported, whereas only a few QTL were found to be stable in different genetic backgrounds and useful for breeding. The most stable QTL were obtained from the Chinese wheat varieties Sumai 3 and Wangshuibai [3]. However, poor agronomic performance and the frequent occurrence of genetic linkage drag make them less suitable donors of resistant genes [4]. Moreover, the genetic and molecular basis of the quantitative FHB resistance is still poorly understood.

Recent studies on the mode of *Fusarium* spike colonisation have revealed that the pathogens use a specific arsenal of virulence factors which are essential in nearly all phases of the disease making them interesting targets for novel resistance strategies. Trichothecene toxins, such as deoxynivalenol (DON), and hydrolytic enzymes, such as subtilisin-like and trypsin-like proteases, are two virulence factors that were found to occur during almost the entire course of disease [5,6]. DON was found to be produced in the fungal infection structures already during the initial penetration of floret tissues [7,8]. The reason for this early secretion remains unknown, because the initial infection is symptomless and indistinguishable between susceptible and resistant wheat cultivars in all respects [9]; even the trichothecene-deficient *Fusarium* mutants do not show any restrictions regarding their infectious ability [10-12]. However, already in the second infection phase, DON production gains relevance. It is supposed that the general capacity to prevent protein synthesis makes the toxin an important suppressor of early plant defences [13,14]. For that purpose, DON seems to enable the fungal hyphae to break through the spike rachis node which is the central bottle-neck for both, the initial spread from infected florets into the spike rachis and the reverse direction from the rachis into uninoculated spikelets [10-12,15,16]. During the rachis colonization when hyphae grow vertically [17], the toxin may inhibit the onset of various cell wall reinforcement processes in the vicinity of invading hyphae [18]. At the same time, fungal proteases are likely to participate in the suppression of plant defences by degrading pathogenesis-related (PR) proteins or defence-signalling compounds according to their property to cause proteolytic protein digestion [19-21]. In the spikes of the resistant landrace Wangshuibai the down-regulation of different housekeeping proteins was reported already 6 to 24 h after *F. graminearum* inoculation as a consequence of the secretion of fungal hydrolytic enzymes and toxins [22].

The intercellular spread through the spike rachis is accompanied by lateral hyphae growth to infect uninoculated spikelets. This secondary colonisation is essentially associated with the secretion of DON and proteases which initiate and facilitate necrotrophic intracellular nutrition. The phase is characterized by dramatic changes in the interaction between pathogen and host concerning the respective transcriptomes, secretomes and metabolomes [7,17,23,24], and is often described as switching point from fungal biotrophy to necrotrophy [25]. Increased DON levels were observed 26 [26] to 96 h [27] after infection (hai). In addition, between 48 and 72 hai *F. graminearum* transcripts were found to encode especially degrading enzymes such as proteases [28]. These accumulations were typically linked to increased levels of systemic fungal development and collapsed host cells [29]. Both virulence factors are probably essential for the penetration and mortification of host cells, as *Fusarium* pathogens use cell wall digestion to enter living host cells [30] and DON, in particular, is known to activate plant programmed cell death [31,32]. In summary, DON and proteases have a significant impact on cell wall digestion, protein matrix reduction and damage to starch granules, typically seen in *Fusarium*-infected wheat kernels rendering grain yield unsuitable and unsafe for food, feed or malting purposes [19,33-36].

In order to characterise the transcriptional changes in the resistant cv. Dream compared with the susceptible cv. Lynx, we performed gene expression profiling using the GeneChip® Wheat Genome Array. GeneChip expression data obtained 32 and 72 h after inoculation with *F. graminearum* or, respectively, mock have revealed indications for the presence of two main defence mechanisms in cv. Dream, reflecting a biphasic strategy against FHB disease. One mechanism comprised jasmonate- and ethylene-mediated defence reactions directed against fungal growth and sporulation, while the second mechanism was specifically directed towards fungal mycotoxins and proteases. Quantitative real-time PCR (qPCR) time-course study was applied to analyse the expressions of seven selected anti-virulence gene candidates in the cultivar pairs Dream/Lynx and Sumai 3 (resistant)/Florence-Aurore (susceptible). Observed similarities between the resistant cultivars Dream and Sumai 3 in terms of FHB-responsive up-regulated genes from both described defence mechanisms will be reported.

## Results and discussion

### Identification of FHB-responsive genes in the resistant wheat cultivar Dream

Transcript abundances in the *F. graminearum* (FHB)-inoculated and mock-inoculated wheat cultivars Dream (resistant) and Lynx (susceptible) were measured and compared using the Affymetrix GeneChip® Wheat Genome Array. The general disease progression was examined on single-floret inoculated samples that were collected 32 and 72 hours after inoculation (hai). All measurements were performed with three biological replicates. For each timepoint the four GeneChip datasets were compared to identify differentially expressed genes involved in the different aspects of the inoculation response. Table 1 lists all comparisons with the respective numbers of differentially expressed genes.

**Table 1:** Number of genes differentially expressed after comparisons of wheat GeneChip datasets at 32 and 72 h after inoculation (hai)

**GeneChip datasets compared**		** Number of genes**			
	**Timepoint**	**32 hai**		**72 hai**	
	**Regulation**	**Up**	**Down**	**Up**	**Down**
Dream *Fusarium* inoculated - Lynx *Fusarium* inoculated		871	924	1,056	681
Dream *Fusarium* inoculated - Dream mock inoculated		115	450	515	318
Dream mock inoculated - Lynx mock inoculated		972	728	*	*
Lynx *Fusarium* inoculated - Lynx mock inoculated		218	204	*	*

* Differentially expressed genes originated from the three replicates of GeneChip dataset ‘Lynx 72 h after mock inoculation’ were not recorded due to low quality of the microarrays.

A gene set enrichment analysis (GSEA) [37] of the comparisons was conducted to identify relevant functional classes associated to incompatible cv. Dream-*F. graminearum* interactions. Table 2 provides an overview of the nine Gene Ontology (GO) terms that were enriched in those genes found to be significantly up-regulated in the resistant cv. Dream at 32 and 72 h after *Fusarium* inoculation compared with the *Fusarium* inoculated susceptible cv. Lynx. All terms were found to be associated to the disease as the respective represented gene products were neither enriched in the analogous cultivar comparison after mock inoculation nor in the comparison ‘cv. Lynx *Fusarium* inoculated versus cv. Lynx mock inoculated’. No GO terms were enriched in the significantly down-regulated genes at 32 hai while 20 enriched terms were observed at the later timepoint (Table 3). For enrichments at 72 hai associations to the infection with *F. graminearum* were restricted and, thus, were only possible if a GO term was also enriched also at 32 hai.

**Table 2:** **GO terms enriched in genes that were significantly up-regulated in the resistant Dream cultivar at 32 and 72 h after *F. graminearum *inoculation (hai)**

**GO term**	**GO definition**	** 32 hai**^**1)**^		** 72 hai**^**1)**^	
		**FDR value**	**NOM p-value**	**FDR value**	**NOM p-value**
GO:0016165	lipoxygenase activity	0.03	0.00		
GO:0031408	oxylipin biosynthetic process	0.04	0.00		
GO:0009405	pathogenesis	0.10	0.01		
GO:0008610	lipid biosynthetic process	0.20	0.0		
GO:0004867	serine-type endopeptidase inhibitor activity	0.00	0.00	0.08	0.00
GO:0004185	serine-type carboxypeptidase activity	0.05	0.00	0.20	0.01
GO:0009611	response to wounding	0.15	0.00	0.21	0.00
GO:0003755	peptidyl-prolyl cis-trans isomerase activity	0.23	0.01	0.07	0.00
GO:0008233	peptidase activity			0.19	0.01

^1)^ Significance levels for up-regulated GO terms: FDR value (False discovery rate) at < 0.25 (probability that gene set represents a false positive finding); NOM p-value (Statistical significance) at < 0.01 (gene-based permutation test).

**Table 3:** **GO terms enriched in genes that were significantly down-regulated in the resistant Dream cultivar at 72 h after *F. graminearum *inoculation (hai)**

**GO term**	**GO definition**	**72 hai**^**1)**^	
		**FDR value**	**NOM p-value**
GO:0042254	ribosomal chaperone activity	0.00	0.00
GO:0003735	structural constituent of ribosome	0.00	0.00
GO:0006412	translation	0.00	0.00
GO:0043581	mycelium development	0.00	0.00
GO:0022626	cellular component	0.00	0.00
GO:0022627	cytosolic small ribosomal subunit	0.00	0.00
GO:0042973	glucan endo-1,3-beta-D-glucosidase activity	0.00	0.00
GO:0030599	pectinesterase activity	0.03	0.00
GO:0044425	membrane part	0.06	0.00
GO:0015935	small ribosomal subunit	0.07	0.00
GO:0008289	lipid binding	0.08	0.01
GO:0006032	chitin catabolic process	0.08	0.01
GO:0042575	DNA polymerase complex	0.13	0.00
GO:0006260	DNA replication	0.15	0.01
GO:0004568	chitinase activity	0.15	0.01
GO:0015934	large ribosomal subunit	0.16	0.01
GO:0016998	cell wall macromolecule catabolic process	0.17	0.01
GO:0022625	cytosolic large ribosomal subunit	0.17	0.02
GO:0005811	lipid particle	0.20	0.03
GO:0006259	DNA metabolic process	0.21	0.02

^1)^ Significance levels for down-regulated GO terms: FDR value (False discovery rate) at < 0.25 (probability that gene set represents a false positive finding); NOM p-value (Statistical significance) at < 0.01 (gene-based permutation test).

The GSEA provided insides into defence mechanisms that were induced during incompatible interactions (Table 2). At 32 hai an exclusive enrichment was observed for the terms ‘lipoxygenase activity’ (GO:0016165), ‘oxylipin biosynthetic process’ (GO:0031408) and ‘lipid biosynthetic process’ (GO:0008610) including genes, such as lipoxygenases, involved in the plant oxylipin metabolism. Additionally, lipoxygenases genes were also frequent in the term ‘response to wounding’ (GO:0009611). Putative cysteine-rich proteins, such as thionins, were detected in the GO term ‘pathogenesis’ (GO:0009405). Phyto-oxylipins comprising antimicrobial peptides and defence-signalling molecules such as jasmonates, together with cysteine-rich pathogenesis-related (PR) genes indicate an induced antifungal defence mechanism [38].

Plant serine-protease inhibitors were enriched in the GO terms ‘serine-type endopeptidase inhibitor activity’ GO:0004867 and ‘peptidase activity’ GO:0008233, and represented the second class of genes enriched in the term ‘response to wounding’ (GO:0009611). Serine-protease inhibitors as well as genes encoding serine-proteases identified by the term ‘serine-type carboxypeptidase activity’ (GO:0004185) were enriched at both timepoints. These enriched terms represent an induced defence mechanism against pathogen-released proteases which as virulence factors are secreted to modify host proteins [6,39]. On the basis of testing, this defence mechanism as well as the antifungal defence mechanism were found to be central and therefore, will be discussed in more detail later on.

Finally, genes encoding for peptidyl-prolyl cis-trans isomerase (PPIase) proteins (GO:0003755) belonging to the immunophilin superfamily were found to be accumulated. PPIase proteins have general functions in protein folding and protein degradation, and several proteins have shown antifungal properties, similar to PR-genes [40,41].

In the down-regulated genes (Table 3) three GO terms were noticeable with regard to the pathogen presence, while associations to the disease were rather unclear for the other terms. The detected GO terms ‘chitin catabolic process’ (GO:0006032) and ‘chitinase activity’ (GO:0004568) demonstrate the down-regulation of genes which typically facilitate the breakdown of fungal cell walls [38]. Chitinase genes have shown to exhibit an enhanced resistance against *F. graminearum*, in barley [42] while in the grains of Emmer wheat (*T. dicoccum*), a progenitor of bread wheat (*T. aestivum*), a similar down-regulation of chitinase genes was observed and discussed as a direct impact of *F. graminearum* signals [43]. Finally, the term ‘mycelium development’ (GO:0043581) comprises 10 *F. graminearum* genes, belonging to a set of 69 *Fusarium* genes which were previously found to be present on the Affymetrix Wheat GeneChip® [44]. As these genes are putatively associated to the progression of the fungal mycelium, their enrichment amongst down-regulated genes might reflect traces of an impaired fungal growth in the resistant Dream cultivar.

**Figure 1:**
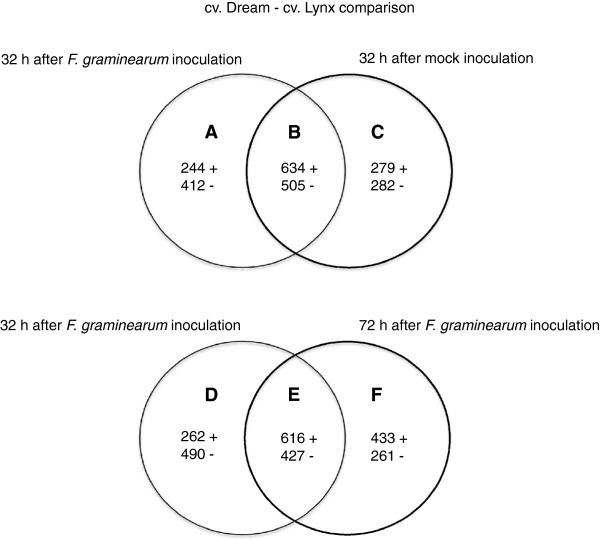
**Venn-diagrams of genes differentially expressed in cv. Dream after treatment and timepoint comparisons.** (Sections **A-C**) Treatment comparison at 32 h after inoculation (hai) between 1,795 genes differentially expressed after ‘cv. Dream *Fusarium* inoculated versus cv. Lynx *Fusarium* inoculated’ and 1,700 genes differentially expressed after ‘cv. Dream mock inoculated versus cv. Lynx mock inoculated’. The Venn-diagram shows the numbers of differentially up- or down-regulated (+/−) genes that were assigned to the following categories of transcript occurrence: (Section **A**) FHB-responsive genes (656) were assumed to reflect induced cv. Dream-controlled differences between both cultivars as they were not differential expressed in the mock-inoculated controls of both cultivars and in the susceptible cv. Lynx after FHB treatment. (Section **B**) Genotype-specific genes (1,139) that were differentially expressed upon both treatments in cv. Dream, but not in cv. Lynx. (Section **C**) The remaining genes (561) were assumed to represent the genetic background of the Dream cultivar as they were also found to be differentially expressed in the absence of FHB-inoculation. For the timepoint 72 hai a corresponding categorisation could not be done due the low quality of the microarrays of the mock treated samples from the Lynx cultivar. (Sections **D-F**) Timepoint comparison for 1,795 genes differentially expressed after ‘cv. Dream *Fusarium* inoculated versus cv. Lynx *Fusarium* inoculated’ at 32 hai in reference to 1,737 genes differentially expressed after the analogous comparison at 72 hai. The Venn-diagram shows the numbers of differentially up- or down-regulated (+/−) genes at the certain timepoints: (Section **D**) Genes found to be differentially expressed in the cv. Dream exclusively at the timepoint 32 hai (752); (Section **E**) at both timepoints (1,043); and (Section **F**) exclusively at the timepoint 72 hai (694).

A comparison was performed between the ‘cv. Dream *Fusarium* inoculated versus cv. Lynx *Fusarium* inoculated’ expression data and the analogous expression data from the mock inoculation (Table 1), in order to address expression changes in the resistant cv. Dream associated with the fungal attack. At 32 hai, the genes differentially expressed in cv. Dream could be separated into genes that were differentially expressed to higher levels or were only present after pathogen attack (Figure 1 section A), after both treatments (Figure 1 section B), and only after control treatment (Figure 1 section C). The genes only present in response to FHB were categorised as ‘FHB-responsive genes’. Especially, up-regulated transcripts are likely to represent defences, such as trigger mechanisms or direct antimicrobial activities. Genes with similar expression profiles after both treatments were categorised as ‘genotype-specific genes’ because they were differentially expressed to lower levels or absent in the cv. Lynx spike samples. Up-regulated genes were hereafter discussed as members of a basal defence if their induction has been demonstrated in previous related resistance studies. Finally, a comparison of the genes differentially expressed in cv. Dream at 32 and 72 h after *Fusarium* inoculation was performed to separate expression changes which have been maintained (Figure 1 section E) from those that were exclusive for one of the two timepoints (Figure 1 section D and section F). Genes that are only differentially expressed at 72 hai (Figure 1 section F) were categorised as ‘72 hai-specific genes’. A mapping into one of the two categories ‘FHB-responsive genes’ and ‘genotype-specific genes’ could not be done, due to the low quality of the microarrays obtained from mock treated cv. Lynx samples at this timepoint (Table 1).

The revised dataset contained a total of 2,169 differentially expressed genes after the transcripts likely to represent general cellular processes in cv. Dream were removed. In total 374 of those genes could be assigned to 11 different defence-related classes. The gene classes and assignments were made based on the GSEA results and on information obtained from FHB-related literature. Table 4 gives an overview of the respective number of individual genes which have been assigned to the three categories and the 11 defence-related gene classes. Detailed information on genes that were identified as putative defence-related is provided in Additional files 1, 2 and 3. The GSEA results were contributing to the formation of the functional classes: (i) ‘jasmonic acid (JA) and ethylene (ET) related genes’; (ii) ‘cysteine-rich antimicrobial peptides (AMPs)’ including serine-protease inhibitors; (iii) ‘jasmonate-regulated proteins (JRP)’ comprising a set of strictly FHB inducible genes; (iv) ‘GDSL-lipases’; and (v) ‘proteolysis’ including serine proteases. Based on literature, genes with different direct or indirect antifungal properties were added to the following classes: (vi) ‘peroxidases’; (vii) ‘genes related to cell wall defence’, for example *PGIP1*s (polygalacturonase inhibiting proteins), xylanase inhibitors and glucan endo-1,3-beta-glucosidase precursors, and (viii) ‘secondary metabolism/detoxification’. The remaining gene classes ‘transcription or signalling genes’, ‘miscellaneous defence related genes’ and ‘hormone metabolism’ have been made for the convenience of discussion.

**Table 4:** **Numbers, classes and categories of genes differentially up- and down-regulated (+/−) in the resistant cv. Dream after *F. graminearum *inoculation (hai)**

**Category**	**Gene class**	** 32 hai**		**32 and 72 hai**				**72 hai**^**1)**^		**Gene class**
				** 32 hai +**		** 32 hai -**				
		**+**	**-**	**72 hai +**	**72 hai -**	**72 hai -**	**72 hai +**	**+**	**-**	**Total**
**FHB-responsive genes**^**2)**^										
	JA and ET related genes	5	3	3	0	0	0	-	-	11
	Cysteine-rich Antimicrobial peptides	4	2	1	0	0	0	-	-	7
	Jasmonate-regulated proteins	3	0	0	0	0	0	-	-	3
	GDSL-lipases	1	0	0	0	0	1	-	-	2
	Proteolysis	1	5	2	0	1	0	-	-	9
	Peroxidases	0	1	0	0	0	0	-	-	1
	Genes related to cell wall defence	4	2	4	0	3	0	-	-	13
	Secondary metabolism/detoxification	10	10	1	0	1	0	-	-	22
	Miscellaneous defence related genes	2	6	4	0	0	0	-	-	12
	Transcription or signalling genes	8	10	3	0	7	1	-	-	29
	Hormone metabolism	1	4	0	0	0	3	-	-	8
Defence related (total)		39	43	18	0	12	5	-	-	117
	Others	51	117	23	0	27	2	-	-	220
	Genes that have no information	74	174	33	0	38	0	-	-	319
Total		163	333	81	0	77	2	-	-	656
**Genotype-specific genes**^**2)**^										
	JA and ET related genes	3	3	6	0	4	0	-	-	16
	Cysteine-rich Antimicrobial peptides	3	1	5	0	0	0	-	-	9
	Jasmonate-regulated proteins	0	0	0	0	0	0	-	-	0
	GDSL-lipases	3	2	7	0	2	0	-	-	14
	Proteolysis	2	0	11	0	5	0	-	-	18
	Peroxidases	2	6	2	0	0	0	-	-	10
	Genes related to cell wall defence	0	3	7	0	0	0	-	-	10
	Secondary metabolism/detoxification	5	8	15	0	12	0	-	-	40
	Miscellaneous defence related genes	0	3	4	0	5	0	-	-	12
	Transcription or signalling genes	3	7	17	0	14	0	-	-	41
	Hormone metabolism	0	1	0	0	2	0	-	-	3
Defence related (total)		21	34	74	0	44	0	-	-	173
	Others	40	58	124	0	85	1	-	-	308
	Genes that have no information	37	66	337	0	217	1	-	-	658
Total		99	157	535	0	346	2	-	-	1,139
**72 hai-specific genes**^**2)**^										
	JA and ET related genes	-	-	-	-	-	-	5	0	5
	Cysteine-rich Antimicrobial peptides	-	-	-	-	-	-	3	0	3
	Jasmonate-regulated proteins	-	-	-	-	-	-	0	0	0
	GDSL-lipases	-	-	-	-	-	-	1	0	1
	Proteolysis	-	-	-	-	-	-	4	3	7
	Peroxidases	-	-	-	-	-	-	4	2	6
	Genes related to cell wall defence	-	-	-	-	-	-	1	1	2
	Secondary metabolism/detoxification	-	-	-	-	-	-	20	4	24
	Miscellaneous defence related genes	-	-	-	-	-	-	6	0	6
	Transcription or signalling genes	-	-	-	-	-	-	15	8	23
	Hormone metabolism	-	-	-	-	-	-	4	1	5
Defence related (total)		-	-	-	-	-	-	-	19	82
	Others	-	-	-	-	-	-	100	1	101
	Genes that have no information	-	-	-	-	-	-	270	241	511
Total		-	-	-	-	-	-	433	261	694
**Defence related (overall total)**										**374**
**Differentially expressed genes (overall total)**										**2,169**

^1)^Genes differentially expressed only at 72 hai were not mapped into one of the two categories ‘FHB-responsive genes’ and ‘Genotype-specific genes’, because of restrictions that originated in low quality of microarrays obtained from mock treated cv. Lynx samples at this timepoint (Table 1).

^2)^Numbers of unique genes are listed that were significantly differentially expressed genes (absolute t-value >1.96 and ≥ 2 fold change). Detailed information on the genes assigned to the different defence-related gene classes is provided in Additional files 1, 2 and 3.

### Indications for a Jasmonate-dependent enhancement of FHB resistance in wheat

Indications for the presence of a JA signalling were found in the cv. Dream transcriptome after FHB infection by using GSEA testing. The GO terms ‘lipoxygenase activity’ (GO:0016165), ‘oxylipin biosynthetic process’ (GO:0031408) and ‘lipid biosynthetic process’ (GO:0008610) associated to the oxylipin metabolism were exclusively enriched in the early 32 hai gene expression data (Table 2) indicating that the chloroplastic 13-LOX-branch was induced upon FHB infection. Hormone-like compounds such as JA and methyl jasmonate (MeJA), as well as 13-HPL-derived C6 aldehydes, are characteristic products of this pathway. Some oxylipins generated by the 13-LOX pathway, for example thaumatin-like proteins and phytoalexins, exhibit antimicrobial activities by impairing fungal mycelial growth and spore germination [45,46]. Other oxylipins, such as JA and MeJA are well known to serve important roles in plant defence-signalling by mediating the induction of the expression of some PR-genes [47-50]. Moreover, as 13-LOX oxylipins are substantially produced from cuticle- or cell membrane-associated fatty acids released during the fungal degradation of plant cell walls, they also act as elicitors involved in pathogen recognition [51].

Threeputative *Lox* genes (Ta.13650.1.A1_at, Ta.1967.2.A1_x_at and TaAffx.104812.1.S1_s_at) were FHB-responsive induced at 32 hai (Additional file 1). The transcript Ta.13650.1.A1_at was found to be a homologue of the maize gene *ZmLOX6* (DQ335764) which is a novel chloroplast localized *Lox* gene described as uniquely regulated by phytohormones and pathogen infection [52] (Table 5). The two transcripts Ta.1967.2.A1_x_at and TaAffx.104812.1.S1_s_at showed significant similarity to the barley gene *Hordeum vulgare methyljasmonate-inducible lipoxygenase 2* (U56406) (Table 5). Therefore, both transcripts might encode for one or two putative methyljasmonate (MeJA)-inducible chloroplastic *13-Lox* genes. It was shown that jasmonates regulate their synthesis *via* positive feedback control by inducing the transcription of biosynthesis genes such as *Lox2*[53-55]. It is remarkable that both transcripts were also already induced 24 h after *F. graminearum* inoculation in the resistant spring wheat cv. Sumai 3 [44].

**Table 5:** **BLASTN analyses of selected genes that were differentially expressed during incompatible cv. Dream–*F. graminearum *interactions**

**Probe set**	**Best BLASTN match**				
	**Description**	**Accession No.**	**Origin**	**Lit. **^**a)**^	**e-value**^**b)**^
Ta.1967.2.A1_x_at	Methyljasmonate-inducible lipoxygenase 2	U56406	Barley	^1)^	0.0
TaAffx.104812.1.S1_s_at	Methyljasmonate-inducible lipoxygenase 2	U56406	Barley	^1)^	0.0
Ta.13650.1.A1_at	ZmLOX6	DQ335764	Maize	^2)^	0.0
Ta.1967.1.S1_x_at	Lox2	AJ507212	Barley	^3)^	5e-146
Ta.485.1.A1_at	Lox2 (lox2:Hv:3 gene)	AJ507213	Barley	^3)^	0.0
Ta.22828.2.S1_at	Lox2	GQ166691	Wheat	^4)^	0.0
TaAffx.90316.1.S1_at	ZmLOX2	NM_001112503	Maize		2e-87
Ta.23763.1.S1_at	WCI-2 (Lipoxygenase)	U32428	Wheat	^5)^	0.0
Ta.188.1.S1_at	WCI-1 (Plant disease resistant response gene)	U32427	Wheat	^5)^	0.0
Ta.23967.1.S1_x_at	THI1.1 (alpha-1-purothionin)	X70665	Wheat	^6)^	0.0
Ta.20930.1.S1_at	PRPI-7 (Durum defensin precursor gene)	GQ449377	Wheat	^7)^	3e-108
Ta.28319.1.S1_at	TaTad1 mRNA for defensin	AB089942	Wheat	^8)^	0.0
Ta.21350.2.S1_at	wrsi5-1 (Bowman-Birk type protease inhibitor, putative)	AY549888	Wheat	^9)^	3e-138
Ta.30711.1.S1_x_at	wrsi5-1 (Bowman-Birk type protease inhibitor, putative)	AY549888	Wheat	^9)^	0.0
Ta.7843.1.S1_a_at	Non-specific lipid-transfer protein 4.3 precursor	HVU63993	Barley		0.0
Ta.31.1.S1_at	VER2 (Vernalization-related gene)	AB012103	Wheat	^10)^	0.0
TaAffx.128684.1.S1_at	ZmOPR2 (12-oxo-phytodienoate reductase 1)	AY921639	Maize	^11)^	0.0
Ta.30921.2.S1_at	ZmOPR4 (12-oxo-phytodienoate reductase 4)	AY921641	Maize	^11)^	3e-144

^a)^Published sources: ^1)^[144]; ^2)^[52]; ^3)^[56]; ^4)^[145]; ^5)^[85]; ^6)^[67]; ^7)^[71]; ^8)^[70]; ^9)^[98]; ^10)^[89]; ^11)^[118].

^b)^For BLASTN analyses the threshold for a significant homology (‘Hit’) was set to an e-value ≤ 1e-20, Identity scale >70% according to [141].

Five *Lox* genes were up-regulated after both treatments and, in contrast to the solely FHB dependent induced *Lox* genes, three of them were also expressed at 72 hai (Additional files 2 and 3). Here, except for the transcript Ta.1967.1.S1_x_at, none of the genes could be assigned to a JA-mediated defence based on sequence similarities to published genes (Table 5). Ta.1967.1.S1_x_at, however, a homologue of a barley gene *Lox2* involved in different stress responses [56] (Table 5), was also shown to be active in cv. Sumai 3 upon *F. graminearum* infection [44].

In summary, putative functions regarding defence response mediation were assigned to genes showing FHB-associated expression alterations. Here, all genes were found to be jasmonate and pathogen inducible or were previously identified as being FHB-responsive in cv. Sumai 3 [44]. This is remarkable as the cultivars Dream and Sumai 3 represent entirely different (geographical) origins and resistance levels. Additionally, JA and ET defence-signalling pathways were found to be essentially involved in the high level FHB resistance of wheat cv. Wangshuibai in a recent study and were supposed to mediate the early basal defences at 12 to 24 h after *F. graminearum* infection [57]. However, the contribution of a salicylic acid (SA) signalling towards FHB resistance reported in that study was neither observed in our study nor reported for the cv. Sumai 3 [44]. On the other hand, a continual JA production can be involved in pathogen defence as well [58]. Indications for JA-inducible as well as for a continual PR-gene expression were indeed observed in the cv. Dream and both might contribute to the present FHB resistance (see below).

### A Jasmonate-responsive and non-specific antifungal defence contributes to FHB resistance

The enrichment of genes belonging to the 13-LOX pathway indicates a systemic accumulation of endogenous jasmonates in the resistant cv. Dream as a result of *F. graminearum* infections. It is known that members of the jasmonate family, whose levels increase on pathogen infection, activate a specific set of genes encoding antimicrobial peptides (AMPs) [38,59]. Several cysteine-rich AMPs were found to be up-regulated in FHB infected cv. Dream spikes, which are possible targets of such resistance-related JA signalling, when the two points in time were investigated. The set of identified cysteine-rich AMPs comprises lipid transfer proteins (PR-14), thionins (PR-13), and defensins (PR-12).

Lipid transfer proteins (LTPs) were the most frequently expressed class of AMPs. Three genes were up-regulated independent of the treatment, while two transcripts were up-regulated exclusively 72 h after FHB inoculation. Compared to the other identified cysteine-rich AMPs, most of the LTP genes have shown relatively high fold changes that remained constant at both timepoints (Additional file 1, 2 and 3). BLASTN analyses proved that all present LTP genes encode for putative non-specific lipid-transfer proteins (nsLTPs). Direct antifungal activities and a broad resistance spectrum against biotrophic and necrotrophic fungal pathogens have been reported for various crop species and tissues, notably with nsLTPs [60-62]. The observed antifungal activities also include different *Fusarium* pathogens, such as *F. graminearum* (wheat) and *F. solani* (maize and barley), as well as *F. culmorum* and *F. oxysporum* (onion) [61-64]. Thereby, nsLTP proteins were found to strongly inhibit the growth of fungal mycelia as well as the germination of fungal spores, including the conidiospores of *F. graminearum*[63,64]. Wheat ns-LTPs are generally supposed to play a role in an enhanced non-specific defence response regulated by different hormonal signals, including jasmonates. In particular, constitutively expressed genes are supposed to contribute to non-host resistance [63].

A synergistic activity of nsLTP genes with thionins (PR-13) against *F. solani* and *F. graminearum* was shown in studies on barley, maize and wheat [62,63,65,66]. In fact, two transcript sequences (Ta.23967.1.S1_s_at and Ta.23967.1.S1_x_at) homologous to the wheat thionin gene *THI1.1*[67] were differentially expressed in the cv. Dream after both treatments, but not in the cv. Lynx (Additional file 2). Thionins have a general antimicrobial activity against early conidial germination [68]. In addition, a highly inducible expression was observed in the case of the *Arabidopsis* thionin *Thi2.1* after both fungal infections as well as MeJA treatment leading to an enhanced resistance to *F. oxysporum*[69].

Peptidase inhibitors of the defensin family (PR-12) make up the third class of continual up-regulated AMPs (Additional file 2), represented by homologues of the wheat gene *Tad1*[70] (Ta.28319.1.S1_at) and the defensin precursor *PRPI-7* from durum wheat (*T. durum*) [71] (Ta.20930.1.S1_at) (Table 5). While the antimicrobial activity of defensins requires typically complex synergistic interactions with other AMPs [72], their promoters are potentially interesting candidates for the targeted and tissue-specific expression of PR- and R-genes, particularly for the protection against *F. graminearum* in cereal grains [73].

An induction by jasmonates was reported for most of the defensin genes and some of the putative antifungal defensins are reported to be markers for the presence of JA- and ET-dependent defence-signalling pathways [74]. Indeed, indications for an active ET signalling were found in the FHB-attacked cv. Dream transcriptome as well (see below).

The majority of up-regulated cysteine-rich AMPs in cv. Dream have shown expression values that were independent of the treatment, but were lower or absent in the susceptible cv. Lynx (Table 4, Additional file 1 and 2). It is likely that the majority of these peptides act synergistically in a generalized non-specific defence providing a basal protection. AMPs transcribed at a constant level are known key components of an immediate defence against invading pathogens [75-77], and many proteins that are pathogen-inducible, for example, in leaves were found to be constitutively present in storage tissues, such as seed [38]. Moreover, it is generally assumed that genes involved in the quantitative FHB resistance of adapted European wheat cultivars represent such a defence mechanism [78].

Nonetheless, AMPs can also be part of an induced plant defence [79]. In FHB-treated cv. Dream spikes only nsLTP genes were up-regulated in response to the disease (Additional file 1). Among these Ta.7843.1.S1_a_at (Table 5) seems to be an interesting resistance candidate, as the gene combines a general high antifungal property with considerable fold change expression ratios at both timepoints (Additional file 1). Moreover, the putative defensin gene *PRPI-7* (Table 5) might be a relevant finding as well due to its possible utilization in a resistant strategy aiming at over-expressions of pathogen-inducible promoters to directly target the infection sites or the most vulnerable tissues [71]. Such an approach becomes even more interesting with the recent observation that the biotrophic life form of *F. graminearum* persists in all colonized tissues [17]. Living host cells form a zone surrounding the most advancing hyphae and could be targets for such an approach as they allow a continuous supply with antifungals onto the intercellular hyphal tips.

### Identification of FHB-responsive jasmonate-regulated proteins

Taking into account the observations on the Dream and Sumai 3 cultivars it can be hypothesised that a FHB-responsive JA signalling is active from 24 to 32 hai covering the presumed phase of the general biotrophic fungal growth, since the switching point to increased necrotrophic nutrition was timed around 48 hai [5,23]. An interesting conformance was observed with the FHB-responsive expressions of genes that encode for jasmonate-regulated proteins (JRP), belonging to the subfamily of mannose-specific jacalin-like lectin containing proteins (mJRL) during that period [80].

Three mJRL genes, TaAffx.7388.1.S1_at, Ta.188.1.S1_at and Ta.31.1.S1_at, were up-regulated in cv. Dream at 32 hai. The first two transcripts are prominent due to the considerable fold change expression ratios of 20.9 and 21.7 (up) (Additional file 1) and their up-regulation exclusively in the FHB treated spikes of cv. Dream. As many mJRL genes are described as strictly inducible defence proteins [80-82], TaAffx.7388.1.S1_at and Ta.188.1.S1_at might be involved in the FHB defence. BLAST analysis showed that all detected putative mJRL genes belong to the mJRP-32 protein subfamily. In general, mJRP-32 genes are specifically induced by JA *via* transcriptional activation and were initially identified in jasmonate-treated barley leaves. The first mJRP-32 gene analysed in detail was the *BGAF* (beta-glucosidase aggregating factor-like protein) gene from maize [82]. The sequence of the transcript TaAffx.7388.1.S1_at shows similarities to another maize *BGAF* gene (NM_001111494) [83] as well as to the wheat gene *Ta-JA1* (AY372111). Although detailed knowledge on the defence function of mJRP-32 proteins is still to be gained, a broad resistance spectrum has already been observed. One prominent example is the *Ta-JA1* gene that encodes a modular *BGAF*-related protein with a proven broad-spectrum resistance to infections by bacterial, fungal and viral pathogens in transgenic tobacco plants [84]. All currently known mJRP-32 genes come from *Poaceae* and share important traits separating them from other mJRLs, for example their exclusive, tissue-specific induction *via* jasmonates and their single-copy status. However, notably due to their strict tissue-specific expressions, mJRP-32 genes are not supposed to be orthologous, although the proteins share numerous common features [82]. An mJRP-32 gene expressed in spike tissues has not been reported so far. For this reason and due to its FHB-responsive high level induction, a separate study should reveal whether the TaAffx.7388.1.S1_at gene represents a new spike-specific member of the mJRP-32 family.

In addition to *Ta-JA1*, the *Poaceae* JRP-32 family comprises three other wheat genes: *Ver2* (Table 5), *WCI-1* (Table 3) and *Hfr-1* (AF483596). In the present work, the wheat chemically-induced gene *WCI-1* and the vernalisation-related gene *Ver2* were up-regulated in cv. Dream upon *F. graminearum* infection. *WCI-1* (Ta.188.1.S1_at) was characterised as a plant disease resistant response gene that is induced by Benzothiadiazole (BTH) [85]. BTH is a functional analogue to SA which was not successful in reducing the FHB-disease caused by *F. graminearum*[86]. On the other hand, an up-regulation of *WCI-1* upon MeJA application has been reported [87], and the *WCI-1* orthologous pea gene *DIR1* was found to be involved in the resistance to different *Fusarium* pathogens [88]. Due to these contradictory observations further examinations are required to clarify the role of *WCI-1* in FHB resistance.

The up-regulation of the vernalisation-related gene *Ver2* (Ta.31.1.S1_at) upon *F. graminearum* infection is interesting. Indeed, due to the proven specific induction by MeJA, *Ver2* was initially proposed to be involved in a jasmonate mediated plant defence response. However, an induction of expression upon *F. culmorum* infection could not be confirmed and a native *Ver2* induction has so far only been observed in young wheat seedlings during the vernalisation process [89]. Thus, whether the untypical expression of *Ver2* in wheat kernels is associated with FHB resistance, or rather is a side effect caused by jasmonate-signalling remains unanswered at this point.

### An increased ethylene (ET) production contributes to wheat FHB resistance

Ethylene (ET) plays an important role in plant growth and development but it is also known to be involved in the regulation of primary resistance responses [90]. Indications for an increased ET-metabolism in cv. Dream spikes following FHB infection are provided by several up-regulated putative 1-aminocyclopropane-1-carboxylate (ACC) oxidases and GDSL-like lipases genes.

The ACC oxidase, also called the ET-forming enzyme, catalyses, together with the enzyme ACC synthase, the last biosynthetic step to convert ACC into ET. Both enzymes are known to be rate-limiting components in the ET biosynthetic pathway [90]. A total of 10 ACC oxidase genes were either up-regulated or down-regulated in the cv. Dream, mainly in a constitutive manner (Additional file 1 and 2). In fact, the expression of individual ACC oxidase genes is generally frequent and differentially regulated at all times due to developmental changes as well as abiotic and biotic stress factors [90,91].

The occurrence of several GDSL-like lipase genes in the cv. Dream assay further indicates an elevated ET-signalling. GDSL-like lipases were mainly differentially expressed upon both treatments (Additional file 1 and 2). Among the characterised GDSL-like lipases, the genes *GLIP1* and *GLIP2* of *Arabidopsis* are known to play an important role in plant immunity by eliciting local as well as systemic resistance against necrotrophic and hemibiotrophic pathogens. Moreover, GDSL-like lipase transcription was exclusively enhanced by ET, but not by SA or JA [92]. However, none of cv. Dream GDSL-like lipases has shown a sequence homology to the reported resistance candidates from *Arabidopsis*.

It is generally accepted that the plant defence against necrotrophic pathogens is usually regulated by JA and ET while SA plays a major role in the defences against biotrophic pathogens [93]. A possible involvement of ET in FHB resistance has also been demonstrated for Sumai 3, based on an up-regulated ACC oxidase gene. Furthermore, an increased resistance against FHB was observed for the susceptible cultivar Y1193 after spraying spikelets with JA as well as ET before and after fungal infection [44]. Different studies in *Arabidopsis*[90] and tobacco [94] have shown that ET and, in particular, the over-expression of certain ACC oxidase genes can extend the symptomless biotrophic phase during hemibiotrophic fungal infections. In addition, it was found that ET can reduce cell death caused by the fungal toxin Fumonisin B_1_ which is produced by several cereal-attacking *Fusarium* species [95].

### Indications for FHB-responsive suppression of fungal virulence factors

In addition to the presence of JA- and ET-mediated general antifungal defences, a second line of defence was found to be based on a FHB-responsive and targeted suppression of relevant *Fusarium* virulence factors, such as proteases and mycotoxins. This defence mechanism was assembled from genes encoding protease inhibitor (PI) proteins (PR-06) and different genes which are proposed to be associated with the detoxification of pathogen-derived mycotoxins. Both, *Fusarium* proteases and mycotoxins take on relevant roles in the fungal pathogenesis and were found to be secreted in nearly all phases of the fungal wheat spike colonisation [9,19,20,22,96].

### Wheat-derived protease inhibitor genes in FHB disease resistance

In the FHB-treated cv. Dream transcriptome, serine PI proteins of the subtilisin-like protease (SLP) superfamily were significant enriched at both timepoints (Table 2), represented by the Go terms ‘serine-type endopeptidase inhibitor activity’ (GO:0004867) and ‘peptidase activity’ (GO:0008233). PI proteins generally feature a high substrate specificity and therefore, it is likely that those genes encode for proteins that specifically bind and impair secreted *Fusarium* SL proteases [97]. Proteases generally cause the proteolytic digestion of proteins *via* the hydrolysation of peptide bonds. *Fusarium* subtilisin-like (SL) and trypsin-like (TL) proteases are released in infected wheat kernels mainly to disrupt host cell membranes during necrotrophic intracellular nutrition. Consequently, defence-related interactions between plant PI proteins and subtilisin-like and trypsin-like proteases of *F. graminearum* and *F. culmorum* have already been proven in the grains of barley [6,24] and ancient emmer wheat (*T. dicoccum*) [43].

In total, five serine-protease inhibitors were differentially up-regulated in cv. Dream (Additional Table 1,2 and 3). Two transcripts were functionally annotated to the Bowman-Birk inhibitor (BBI) family based on sequence homologies to the *WRSI5* gene (Table 5). *WRSI5* was described as a salt-responsive gene with a suggested role in regulating plant growth [98]. Among the remaining transcripts, Ta.2632.2.S1_x_at and Ta.2632.3.S1_x_at were up-regulated in response to FHB at 32 hai, while Ta.22614.1.S1_at was regulated solely at 72 hai.

The Ta.22614.1.S1_at gene was selected for qPCR analysis because of its relativelyhigh and FHB-responsive fold change (9.49 up) at 72 hai (Additional file 3). A possible expression at 32 h after Fusarium inoculation was not reliably determined due to missing expression data from two of the three biological replicates. The Ta.22614.1.S1_at expression was measured with an inoculation time-courses of two cultivar pairs: first, the winter cultivars Dream and Lynx described as moderately resistant and susceptible (time-course of 8 to 96 hai); second, the spring cultivars Sumai 3 and Florence-Aurore described as resistant and susceptible (time-course of 0 to 336 hai). Sumai 3 and Florence-Aurore, in particular, were found to represent the extremes of spring wheat responses to *Fusarium* spread [99,100].

**Figure 2:**
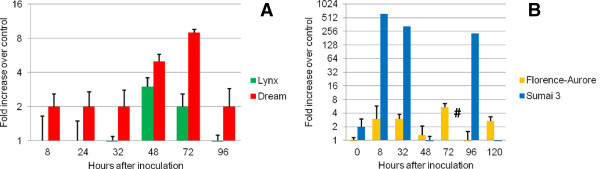
**Expression analysis of the subtilisin-like serine protease inhibitor gene Ta.22614.1.S1_at in two cultivar pairs.** Ta.22614.1.S1_at was analysed as a candidate gene for resistance against fungal-derived proteases. The qPCR time-course experiment was conducted on infected spikes of (**A**) the moderately FHB-resistant cv. Dream and the susceptible cv. Lynx; and (**B**) the FHB-resistant cv. Sumai 3 and the susceptible cv. Florence-Aurore. Fold increases were calculated relative to the internal standard gene (ubiquitin) and a water treated control sample (mock) of the respective sampling time using the comparative C_t_-Method. Columns represent average induction (+SE, n = 3) plotted on a 2x logarithmic scale. Hash symbols indicate measurements where no gene expression was observed.

Upon comparing the FHB-responsive transcript induction levels in the cultivars Dream and Lynx, a generally higher induction over control and cv. Lynx samples was observed for cv. Dream for the period between 24 to 96 hai (Figure 2A). As a matter of fact, >4-fold inductions were only obtained at 48 and 72 hai. However, even the 2-fold inductions of earlier and later timepoints were considered relevant due to the strictly suppressed expression in the susceptible genotype (Figure 2A). In the FHB-treated spike tissues of Sumai 3, >600 and >300-fold inductions were already observed between 8 to 32 hai and a third peak of >200-fold was found at 96 hai (Figure 2B). No gene expression was verifiable in spike samples of cultivars Sumai 3 and Florence-Aurore at 336 hai which was the last timepoint (not shown).

In the first instance, the relative induction peak at 72 hai in cv. Dream is consistent with previous observations that endogenous wheat serine-protease inhibitor proteins are not induced until 72 h after *Fusarium* inoculation [101]. In fact, in the period between 48 and 72 hai, during which the necrotrophic nutrition becomes predominant, *F. graminearum* transcripts were found to dominantly encode degrading enzymes such as proteases, lipases and nucleases [29]. A transcript accumulation was even observed in the susceptible genotypes Lynx (Figure 2A) and Florence-Aurore (Figure 2B) particularly in this period, however, to a lower extent than in the respective resistant counterparts. The physiological responses of PIs are furthermore triggered by negative feedback mechanisms [102]. Therefore, the remarkable suppression of Ta.22614.1.S1 in Sumai 3 during this crucial time might be a consequence of the already high transcript abundance and the subsequent induction at 96 hai is assumed to be stimulated by further secreted fungal proteases (Figure 2B).

The early high level activity of Ta.22614.1.S1 until the timepoint 32 hai in cv. Sumai 3 (Figure 2B) is consistent with previous data from Sumai 3 gene expression studies, demonstrating the FHB-responsive expression of several PIs at already 24 hai [44]. In this period, an exclusive induction of the tested serine-protease inhibitor was also observed for the moderately resistant cv. Dream (Figure 2A). Consequently, the early expression of wheat PI genes could be an immediate reaction to early levels of secreted *Fusarium* TL and SL proteases which have been reported for different compatible interactions, amongst others between *F. graminearum* and barley as well as wheat. Here, the activity of fungal proteases has been attested already at 6 to 24 hai, long before a corresponding expression could be observed in kernels [22,28]. In fact, beside their harmful roles during the phase of a necrotrophic intracellular nutrition, fungal proteases were found to be secreted already during the earlier intercellular colonisation of spike rachis, probably to suppress certain plant defence reactions by degrading PR-proteins [15,20,21,96,103].

In this sense, the serine-protease inhibitor Ta.22614.1.S1_at seems to be an interesting resistance candidate as transcript accumulations were present during the early and the later phases of fungal spike colonisation. However, this potential still needs to be confirmed in a further study. Nevertheless, PIs are discussed as candidates for an improved resistance strategy against grain infecting fungal pathogens [19,104] and our results from qPCR and transcriptome analyses do not contradict these considerations.

### Analysis of the detoxification mechanisms in wheat concerning FHB resistance

*Fusarium* proteases and mycotoxins act in a kind of strategic cooperation during spike and kernel colonisation by featuring complementary roles during the host defence suppression and the intracellular colonisation of spikelets. From an economic perspective, *Fusarium* species causing FHB belong to the most important trichothecene producers and DON is a predominant trichothecene toxin produced by these species [105]. Silencing the *Fusarium TRI6* gene down-regulates more than 200 genes involved in the mycotoxin production and results in a reduction of DON production and pathogenicity [8]. Meanwhile, several different plant genes are known to be up-regulated at the transcriptional level in response to either DON treatment or DON production which are thus likely to be involved in the DON-resistance [105].

To analyze the expected impact of a specific mycotoxin defence on the general FHB resistance of cv. Dream, a literature-to-transcriptome approach was used. Known toxin resistance-related genes from wheat and barley were checked for homologous genes on the wheat array and their respective expression profiles in the cultivars Dream and Lynx. A diverse set of 26 wheat genes could be identified as possible members of a general detoxification mechanism. Those genes are listed in Table 6, including the respective literature sources. Within this set, 12 genes originate from a study of trichothecene-induced gene expression in barley [106]. Screening the expression patterns of those 26 genes in the cv. Dream vs. cv. Lynx microarray data revealed for all genes similar expression patterns. They were exclusively expressed or induced in *Fusarium* treated samples collected 72 h after infection. Moreover, they were also up-regulated in both genotypes and, in addition, they were up-regulated in both genotypes and the level of up-regulation was higher in susceptible cv. Lynx in all cases. However, expression differences between both genotypes never reached a level of statistical significance. Finally, they were not expressed in mock control samples at all, although this observation was not reliable in the case of cv. Lynx samples collected at 72 hai due to the above mentioned restrictions. To analyse the observed congruities in more detail and to test whether or not the expression in the susceptible cv. Lynx is just a temporary phenomenon, a selection of six genes representing different functional categories was forwarded to qPCR analysis using the above mentioned inoculation time-courses of the cultivar pairs Dream vs. Lynx and Sumai 3 vs. Florence-Aurore.

**Table 6:** **Presumed trichothecene-responsive genes with similar expression pattern during incompatible cv. Dream–*F. graminearum *interactions**

**Probe set**	**Class**^**a)**^	**Description**	**Accession No.**^**b)**^	**Lit.**^**c)**^
Ta.12887.1.S1_at	Trichothecene	UDP-glucosyltransferase HvUGT13248	GU170355	^1); 2); 3); 10); 12)^
Ta.1811.1.S1_at	Trichothecene	UTP-glucosyltransferase	EU496513	^3); 5)^
Ta.23272.1.S1_at	Trichothecene	TaUGT3 (UDP-glucosyltransferase protein)	FJ236328	^3)^
Ta.8495.1.A1_at	Trichothecene	UDP-glucosyltransferase	AJ438338	^3)^
Ta.23340.2.S1_at	Trichothecene	cv. Sumai3 UDP-glucosyltransferase	HM133634	^3)^
Ta.22565.1.S1_at	Trichothecene	TaUGT1 (UDP-glucosyltransferase protein)	EU552210	^3); 10)^
"	"	TaUGT2 (UDP-glucosyltransferase protein)	EU568801	
Ta.8232.1.A1_at	Trichothecene	TaPDR1 (pleiotropic drug resistance 1)	FJ185035	^7)^
Ta.6990.1.S1_at	Trichothecene	OsPDR5 (pleiotropic drug resistance 5), putative	FJ858380	^9)^
Ta.9385.3.S1_at	Trichothecene	Putative PDR-like ABC transporter related cluster	AY332479	-
Ta.2793.1.S1_at	Trichothecene	TaMDR1 (MDR-like ABC transporter)	AB055077	^1); 8)^
TaAffx.91779.1.S1_at	Trichothecene	MRP (Multidrug Resistance-associated Protein) ^**d)**^		^4)^
Ta.6621.1.A1_at	"	"		
TaAffx.91779.2.S1_at	"	"		
Ta.28932.1.S1_at	Trichothecene	MRP2 (Multidrug Resistance associated Protein 2)	AF532601	^6)^
Ta.27443.1.S1_at	Trichothecene	MRP3-like ABC transporter		^1); 2)^
TaAffx.12277.1.S1_at	Trichothecene	MATE efflux family protein ^b)^		^1)^
Ta.4165.1.S1_at	Trichothecene	major facilitator superfamily antiporter, putative		^1)^
Ta.8990.1.S1_at	Oxidative burst	Glutaredoxin-like		^1)^
Ta.233.1.S1_at	Oxidative burst	*Waox1a* gene (alternative oxidase AOX)	AB078882	^2)^
TaAffx.81871.1.S1_at	Regulatory	AAA-type ATPase		^1)^
Ta.3902.1.S1_at	Regulatory	AAA-type ATPase family protein		^2)^
Ta.7015.1.S1_at	Regulatory	F-box domain containing protein		^1)^
Ta.5155.1.S1_at	Defence	Putative blue copper binding protein		^1)^
Ta.8040.1.A1_at	Defence	Putative subtilisin-like serine proteinase	XM_003581069	^1)^
Ta.26151.1.A1_at	Defence	Putative subtilisin-like serine proteinase		^1)^
Ta.1207.1.S1_at	General	ZmOPR1 (12-oxo-phytodienoate reductase 1)	NM_001112429	^1); 11)^
Ta.19609.1.S1_at	General	cytochrome P450		^2)^
Ta.8017.1.S1_at	Unknown	hypothetical protein		^2)^

^a)^Class association as obtained from respective literature sources.

^b)^Gene annotations were updated by BLASTN analysis in the NCBI database, otherwise published annotations were considered.

^c)^Published sources: ^1)^[106]; ^2)^[116]; ^3)^[4]; ^4)^[146]; ^5)^[109]; ^6)^[147]; ^7)^[108]; ^8)^[110]; ^9)^[107]; ^10)^[115]; ^11)^[118]; ^12)^[117].

^d)^Predicted MRP gene sequence contains EST cluster: CA732909, BJ309016 and BJ303163 [146].

The analysed genes associated with DON detoxification are *TaUGT3* (UDP-glucosyltransferase protein) and a homologue of the barley UDP-glucosyltransferase gene *HvUGT13248*. Genes that are supposed to be involved in the resistance to DON accumulation are *TaPDR1* (pleiotropic drug resistance 1) and *TaMDR1* (MDR-like ABC transporter gene). As representatives of the functional categories “defence-related” and “general” a further putative serine-protease gene and a 12-oxophytodienoate reductase gene were included (Table 6).

**Figure 3:**
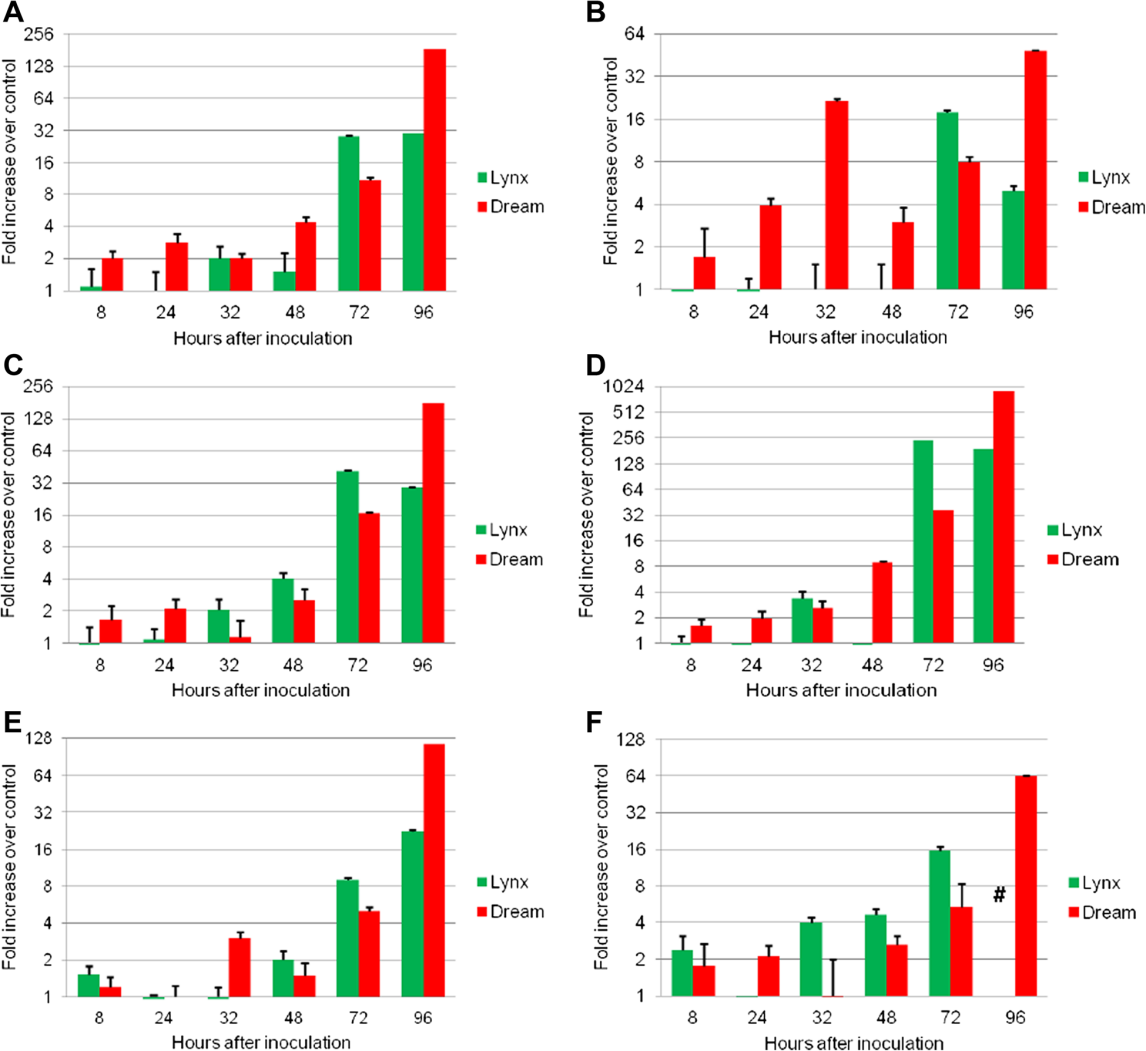
**Analysis of gene expressions in the moderately FHB-resistant cv. Dream and the susceptible cv. Lynx.** The qPCR time-course experiment was used to determine the expressions of six genes selected due to their proposed association to resistance against the *F. graminearum*-derived toxin deoxynivalenol (DON). Fold increases were calculated relative to the internal standard gene (ubiquitin) and a water treated control sample (mock) of the respective sampling time using the comparative Ct-Method. Columns represent average induction (+SE, n = 3) plotted on a 2x logarithmic scale. Hash symbols indicate measurements where no gene expression was observed.

The qPCR data for the winter wheat cultivars Dream vs. Lynx (Figure 3A- 3D) showed similar expression patterns for all tested genes as did the microarray experiments. Consequently, a temporary and higher induction peak was found for Lynx at 72 hai compared to Dream. On the other hand, the transcripts of all tested genes peaked at 96 hai in the cv. Dream samples, while Lynx revealed suppressed or consistent inductions. In addition, a >4-fold induction was already observed before 72 hai for most of the cv. Dream alleles and the expressions were showing a general and increasing trend towards the peak at 96 hai (Figure 3A-3D). Such a maximum induction at 96 hai has likewise been observed for the DON resistance candidate gene *PDR5* (*pleiotropic drug resistance5*)*-like* in infected spikes of the Chinese landrace Wangshuibai [107] which represents one of the most important genetic resources for FHB and DON resistance [99]. Like the analysed genes *TaPDR1* and *TaMDR1* (Figure 3A and 3B), *PDR5-like* is like a plasma membrane ABC transporter which co-segregates with the DON resistance QTL Qfhs.ndus-6BS from cv. Wangshuibai [107].

**Figure 4:**
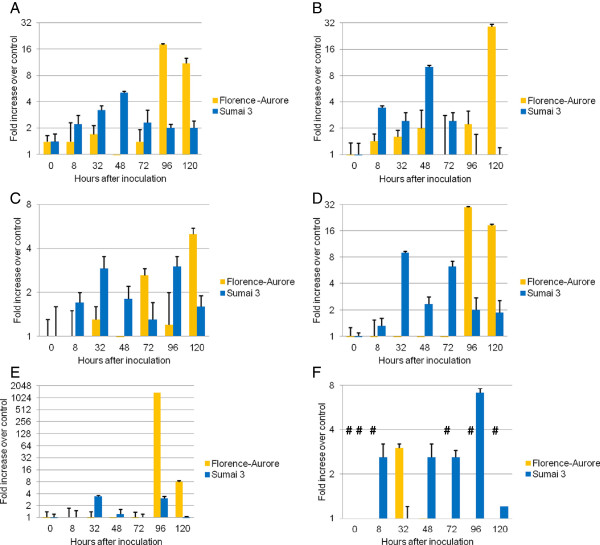
**Analysis of gene expressions in the FHB-resistant cv. Sumai 3 and the susceptible cv. Florence-Aurore.** The qPCR time-course experiment was used to determine the expressions of the six genes selected due to their proposed association to resistance against the *F. graminearum*-derived toxin deoxynivalenol (DON). Fold increases were calculated relative to the internal control gene (ubiquitin) and a water treated control sample (mock) of the respective sampling time using the comparative Ct-Method. Columns represent average induction (+SE, n = 3) plotted on a 2x logarithmic scale. Hash symbols above the bars indicate measurements where no gene expression was observed.

In the cultivar pair Sumai 3 vs. Florence-Aurore the *Fusarium*-induced expression levels obtained for the UDP-glucosyltransferase (UGT) and ABC transporter genes were showing typical curve characteristics in cv. Sumai 3 samples; starting with a low level induction at 8 hai, followed by a consistent increase up to the peak at 32 or 48 hai and showing a continuous downtrend thereafter (Figure 4A-4D). In contrast, considerable inductions for the susceptible cv. Florence-Aurore did not appear until 96 to 120 hai (Figure 4A-4D). Interestingly, both UGT genes show induction peaks at 32 hai in cv. Sumai 3 while both ABC transporter genes peak at 48 hai. Deviating induction patterns were observed for the representatives of the functional categories “defence-related” and “general” (Figure 4E and 4F). For all tested genes no expressions were measured from samples collected at 336 hai (not shown).

In summary, the expression profiles of genes related to detoxification in both resistant cultivars were following an early beginning steady-state model, providing transcript levels during the reported period of increased DON accumulations between 36 and 144 hai [5,13,106]. In contrast, both susceptible cultivars showed typically late and temporary inductions. Our observations for the expressions of UGT and ABC transporter genes in cv. Sumai 3 are furthermore in accordance with expression patterns previously observed for the ABC transporter gene *TaPDR1*[108] as well as the UGT gene *TaUGT3*[4] in FHB-treated spike samples of cv. Wangshuibai.

The *TaPDR1* gene is a member of the ATP-binding cassette (ABC) protein superfamily and has been identified in cv. Wangshuibai due to its strong up-regulation upon DON treatment as well as *F. graminearum* inoculation. After fungal infection, the relative amount of *TaPDR1* transcripts increased in Wangshuibai at 48 hai [108]. The function of *TaPDR1* in FHB resistance is proposed to be DON-related because gene expressions were found to peak after 6 to 12 h of DON-inoculation and declined slowly thereafter. In addition, a late expression peak was observed for the susceptible cv. Alondra [108] similar to our observations in the susceptible cv. Florence-Aurore (Figure 4A). The general role of PDR transporters in the resistance to antifungal drugs was first characterized in yeast (*S. cerevisiae*) and a particular function in DON resistance was confirmed based on a yeast mutant carrying a knockout variant of the PDR5 transporter gene resulting in a non-natural hypersensitivity to DON [109].

The second analysed transporter gene *TaMDR1* was initially isolated from wheat root apices as being induced by aluminium toxicity [110]. However, *TaMDR1* was up-regulation together with *TaPDR1* in cv. Wangshuibai and, thus, was supposed to be involved in DON resistance as well [108]. In fact, our time-course qPCR expression data were able to reveal that both genes show similar expression profiles upon *Fusarium* infection in the resistant cultivars Dream (Figure 3A and 3B) and Sumai 3 (Figure 4A and 4B), respectively. Although genotype-specific differences were present, the observed similar expression patterns indicate a possible trichothecene-responsive up-regulation for *TaMDR1* as well. Using nullisomic-tetrasomic wheat lines, we have also located the *TaMDR1* allele on chromosome 5A where *TaPDR1* had already been placed before [108]. These observations may reflect a common mechanism of transcriptional co-regulation for both genes. In general, there is accumulating evidence that gene order in eukaryotic genomes is not completely random and that pathogen-responsive as well as other genes with similar expression levels tend to be clustered within the same genomic neighbourhoods [111]. In fact, for *TaPDR1* it was discovered that the gene expression is not induced by JA, SA and abiotic stress factors but by decreasing concentrations of Al^3+^ and free [Ca^2+^. This mode of regulation was also reported for the *TaMDR1* gene due to its general induction by Al injury in wheat roots [110]. Both toxicities activate plant programmed cell death *via* an oxidative burst and both inhibit calcium channels of plasma membranes which causes a decrease of the intracellular second messenger [Ca^2+^[31,112,113]. In order to explain the commonalities we have found between both ABC transporters in terms of induction, we suggest that DON induces the expression of *TaPDR1* and *TaMDR1* indirectly *via* decreased levels of [Ca^2+^. Whether *TaMDR1* thus has a similar relevance for the detoxification process as can be suggested for *TaPDR1*, still needs to be proven in a further study.

Two UGT genes supposed to be involved in the DON detoxification were analysed with qPCR. Quite a few of the plant UGTs are related to disease resistance where they play important roles in the detoxification of exogenous compounds, for example fungal metabolites such as DON [114]. BLASTN analysis revealed the homology between the transcript Ta.23272.1.S1_at and the *TaUGT3* (FJ236328) gene which had originally been cloned from cv. Wangshuibai [4]. Ta.12887.1.S1_at has revealed a significant full length sequence homology to the barley UGT gene *HvUGT13248*[115] (Table 4). Both genes have displayed the respective characteristic qPCR expression profiles for cvs. Dream (Figure 3C and 3D) and Sumai 3 (Figure 4C and 4D) as described above. However, higher induction levels were observed for the putative *HvUGT13248* gene when compared to *TaUGT3*.

At the first instance, the wheat gene *TaUGT3* was the most interesting candidate since it was suggested to be an efficient candidate gene for improving DON resistance [4]. However, our expression data are in accordance with recent observations which have demonstrated that *HvUGT13248* can protect yeast from DON by converting it to DON-3-glucoside while *TaUGT3* was not able to convert DON [115]. In addition, with our observations in the cultivars Dream and Sumai 3, *HvUGT13248* has demonstrated relevant activities in a number of FHB-treated wheat cultivars as well as in barley, indicating that it be might of general relevance. *HvUGT13248* (Ta.12887.1.S1_at) and also *TaUGT3* (Ta.23272.1.S1_at) were detected as DON resistance candidates in DON inoculated spikes of cv. Wangshuibai in a gene expression study using the Affymetrix Wheat Gene-Chip® [4]. Moreover, BLASTN analysis could demonstrate that *HvUGT13248* has also been identified as DON resistance related gene (TA/EST accession TA88294_4565) in wheat DH-lines carrying the major FHB resistance QTL *Fhb1* from cv. CM82036 [116] as well as in two related barley transcriptome studies (Barley1GeneChip® probe Contig13248_at) [106,117]. Finally, the gene *HvUGT13248* appears to be a remarkable candidate gene for FHB resistance. It is considered relevant for a promising strategy to improve FHB resistance not only in wheat but also other cereal species.

As representative for the functional category “general”, the expressions of a putative wheat gene encoding for a 12-oxophytodienoate reductase was analysed (Table 4). Ta.1207.1.S1_at was functionally characterised by significant homology to the maize 12-oxo-phytodienoate reductase gene *ZmOPR1* (Table 6). The homologous barley gene (Contig6194_s_at) was previously found to respond to pathogen-derived trichothecene accumulation [106]. In addition to Ta.1207.1.S1_at, two more putative OPR genes were identified as up-regulated in response to FHB: the gene *ZmOPR2* (Table 5) and the gene *ZmOPR4* (Table 5). All three genes are putative wheat homologous of the OPR I group members which preferentially catalyse the formation of the natural JA precursor 12-oxo-phytodienoic acid (OPDA) [118].

In our qPCR analysis, the *ZmOPR1* homologue Ta.1207.1.S1_at has shown a FHB-associated induction at 32 hai which was common for both the resistant genotypes (Fig 3E and 4E). This might indicate a rapid and transient up-regulation of Ta.1207.1.S1_at. In fact, the genes *ZmOPR1* and *ZmOPR2* have demonstrated a transient induction upon *Fusarium verticillioides* infection in maize [118]. A similar rapid and transient up-regulation caused by a variety of environmental cues including hydrogen peroxide (H_2_O_2_) was observed for the Ta.1207.1.S1_at homologous gene *OsOPR1* (EU146300) in rice [119]. DON is known to induce the transient accumulation of H_2_O_2_ as the most stable compound involved in oxidative burst [31,32]. Indeed, yeast studies indicate detoxifying functions for OPRI enzymes [118].

### Indications for a complex crosstalk between fungal and plant proteases and their inhibitors during FHB defence

The putative wheat serine protease gene (Ta.8040.1.A1_at) belongs to the subtilisin-like protease family and was initially detected as a gene (Contig22733_at) that strictly responds to pathogen-derived trichothecene accumulation in barley [106] (Table 6). In addition, serine proteases were found to be enriched in the cv. Dream transcriptome upon FHB treatment and were annotated to the GO term ‘serine-type carboxypeptidase activity’ (GO:0004185) (Table 2). An early Ta.8040.1.A1_at expression was found for cv. Sumai 3, here, exclusive and equal >2-fold inductions were present at 8, 32 and 72 hai (Figure 4F). At 96 hai, both resistant cultivars showed the highest induction level, in cv. Dream even with a peak of >60-fold, while at this timepoints no expressions were found in the susceptible cultivars (Figure 3F and 4F). An opposing effect was observed at 32 hai, when exclusive expression was observed for both susceptible wheat cultivars, while no expression was detectable in the resistant ones (Figure 3F and 4F).

As proteolytic and protein-binding enzymes proteases feature important functions for the selective breakdown of regulatory proteins and several plant proteases have been linked to defence responses [120]. Although many questions remain unanswered concerning their mode of action, there is evidence that plant proteases, in particular subtilisin-like proteases, are involved in the crosstalk between pathogen and host. In this context, a defence-counter-defence mechanism was observed between the plant-pathogen interaction tomato/*Phytophthora infestans*, in which both, host and pathogen are supposed to release specific sets of proteases and protease inhibitors mutually impairing each other [39,121]. Moreover, such counter-defence mechanism is supported by the assumption of a strong co-evolution between proteases and protease inhibitors which are mutually released during a pathogen-host interaction [122]. It is interesting in this context, that proteases as well as protease inhibitors were enriched in the transcriptome of the resistant cultivar Dream upon *F. graminearum* infection (Table 2). Regarding the role of the reported plant proteases involved in DON resistance (Table 6), we suggest that they do not act in response to a DON accumulation but rather in response to a *Fusarium* protease*-*rich environment as *Fusarium* proteases appear together with mycotoxins during spike rachis and kernel colonisation. In addition, a specific function within a detoxification mechanism has yet not been described for plant proteases.

## Conclusions

Our transcriptome study provides evidence for the existence of a biphasic defence reaction against FHB in wheat. Jasmonate and ethylene regulated non-specific antifungal protections are supplemented by host gene networks associated to the accumulation of *F. graminearum*-derived trichothecenes and subtilisin-like proteases. Using a literature-to-transcriptome approach, 26 genes described as related to DON resistance were identified due to analogies in their microarray expression profiles which hence, may belong to a detoxification pathway that is active in different resistant wheat cultivars as well as in barley. Our qPCR expression analyses of seven wheat genes associated with the suppression of fungal virulence factors have demonstrated similar FHB-responsive inductions in the cultivars Dream and Sumai 3. Moreover, an earlier first induction and a steady-state level of expression were found to be associated with FHB resistance, while FHB-responsive gene expression in susceptible cultivars was typically late and temporary.

These results will help not only to understand changes in overall gene expression in wheat during *Fusarium* infection, but will also help to identify potential targets for development of disease control strategies. In fact, genes interesting for further investigations in this direction were identified in both wheat defence mechanisms. These are, nsLTP (Ta.7843.1.S1_a_at), defensin (Ta.20930.1.S1_at) and mJRP (TaAffx.7388.1.S1_at) genes as well as the PDR-transporter gene *TaMDR1* (Ta.2793.1.S1_at), the UGT gene *HvUGT13248* (Ta.12887.1.S1_at) and the putative serine-protease inhibitor gene Ta.22614.1.S1_at. The last three genes have shown regulations in response to FHB in the cultivars Dream and Sumai 3. In general, the identification of resistance candidate genes that are commonly active in different resistant wheat and barley cultivars is an important result with regard to the development of novel strategies against FHB severity and grain toxin contamination.

## Methods

### Plant and fungal material, inoculations and sampling

*Plant material*: Four wheat genotypes with contrasting levels of FHB resistance were used in this study: the German cultivar Dream (Disponent/Kronjuwel//Monopol/3/Orestis), the British cv. Lynx (CWW-44442-64/Redezvous), the Chinese cv. Sumai 3 (Funo/Taiwan wheat) and the French cv. Florence-Aurore (Florence/Aurore). The winter wheats Dream and Lynx are moderately resistant and susceptible, respectively [100] and inoculated samples were used for both microarray analysis and quantitative real-time PCR (qPCR) based expression analysis. The spring types Sumai 3 and Florence-Aurore are highly resistant and highly susceptible, respectively [99]; and inoculated samples were solely used for qPCR expression analyses.

*Inoculum production*: Macroconidia of the single-spore *F. graminearum* isolate ‘IFA 65’ (IFA Tulln, Austria) were grown on synthetic nutrient agar medium ‘Spezieller Nährstoffarmer Agar (SNA)’ [123] at 20 °C under cool-white and near-UV light illumination. After seven days macroconidia were collected by centrifugation and washed in double-distilled water. For the inoculations 10 ml stock solutions (1x10^5^ macroconidia ml^-1^) of the inoculum were stored at −80°C until use.

*Inoculation and sampling*: Dream and Lynx wheat plants were grown in the greenhouse. After vernalisation at 4°C for eight weeks with a 16/8 h day/night light regime, plants were cultivated at day/ night temperatures of 22/18°C with a photoperiod of 16/8 h (day/night). At early anthesis single floret inoculation with the *F. graminearum* strain ‘IFA 65’ was carried out by pipetting 10 μl of the fungal suspension (5 x 10^4^ macroconidia ml^-1^) between the palea and lemma of each floret [124]. Control (mock) plants were inoculated with distilled water instead of the macroconidia suspension. Eight florets per spike were inoculated. Greenhouse day temperature was increased to 24°C to ensure optimum infection conditions. Tissues of inoculated florets (lemma, palea) and a part of the attached rachis of Dream and Lynx spikes were collected. Six plants per genotype/treatment/timepoint were sampled. Samples were immediately frozen in liquid nitrogen and stored at −80°C. For the microarray analysis three replications were made for each inoculation treatment and samples were collected at 32 and 72 h after inoculation (hai). For the qPCR analysis samples were collected at 8, 24, 32, 48, 72, and 96 hai.

Sumai 3 and Florence-Aurore wheat plants were grown under open air conditions. At early anthesis, spikes were spray inoculated with 2 ml of the *F. graminearum* macroconidia suspension (1 x 10^5^ macroconidia ml^-1^) or distilled water (mock inoculation) according to [125]. For qPCR analysis whole spikes (one spike per single plant) of treated cv. Sumai 3 and cv. Florence-Aurore plants were collected at 0, 8, 32, 48, 72, 96, 120 and 336 hai. Four plants per genotype/ treatment/ timepoint were sampled. All samples were immediately frozen in liquid nitrogen and stored at −80°C.

### RNA extraction and cDNA synthesis

For cv. Dream and cv. Lynx, floret tissue of six wheat heads per genotype, treatment and sampling timepoint were pooled prior to RNA extraction in order to reduce the biological variation between the samples. Accordingly, for cv. Sumai 3 and cv. Florence-Aurore spike tissue of four wheat plants per genotype, treatment and sampling timepoint were pooled prior to RNA extraction.

Total RNA was extracted from fine ground samples using the guanidinium thiocyanate-phenol-chloroform method as described by [126]. Subsequently, a DNase (DNase I, RNase-free, Fermentas) digest was performed according to manufacturer’s instructions. RNA was further purified using phenol-chloroform extraction [127]. RNA quantity and quality were evaluated using ND-1000 spectrophotometer (NanoDrop) measurement and agarose (NEEO Ultra Qualität, Roth, 1.5%) gel electrophoresis. cDNA was synthesised with 1.2 μg total RNA and 0.5 μg oligo(dT)_18_ primers using the RevertAid^TM^ H Minus First Strand cDNA Synthesis Kit (Fermentas, Germany) according to manufacturer’s instructions. cDNA quantity and quality were evaluated using ND-1000 spectrophotometer (NanoDrop, USA) measurement.

### Microarray assay

The Affymetrix Wheat Genome GeneChip® Array (Affymetrix Inc., USA) was used to measure the gene expression changes within the bulked RNA samples of cv. Dream and cv. Lynx. RNA labelling and microarray hybridisation were performed according to the Affymetrix technical manual [128] at the Max Planck Institute for Terrestrial Microbiology, Marburg, Germany. The following wheat samples were analysed (1) cv. Dream, *F. graminearum*-inoculated, 32 hai; (2) cv. Dream, mock-inoculated, 32 hai; (3) cv. Dream, *F. graminearum*-inoculated, 72 hai; (4) cv. Dream, mock-inoculated, 72 hai; (5) cv. Lynx, *F. graminearum*-inoculated, 32 hai; (6) cv. Lynx, mock-inoculated, 32 hai; (7) cv. Lynx, *F. graminearum*-inoculated, 72 hai; and (8) cv. Lynx, mock-inoculated, 72 hai. Three biological replications per genotype/treatment/timepoint were performed. Gene expression intensities were extracted from the scanned GeneChip images, data analysis was performed using the Bioconductor packages "affy", "gcRMA" and "limma" [129] within the R environment. Data were preprocessed using the affy package [130] and normalised by the gcRMA method [131]. The limma package [132] was used for the analysis of differentially expressed genes. Genes with an absolute t-value >1.96 that were at least two-fold regulated were selected as differentially expressed genes. Such genes were assigned as ‘induced’ or ‘repressed’.

To identify enriched gene ontology terms, a gene set enrichment analysis was carried out using the GSEA (Gene Set Enrichment analysis) platform [133]. The gene ontology annotations were received by using Blast2GO. Significant enriched gene sets were selected based on a FDR < 25% and a gene set size > 15.

The following publicly available databases were considered for functional annotations: PLEXdb (Gene expression resource for plants and plant pathogens) [134], NCBI (National Center for Biotechnology Information) [135], RGAP 6.1 (Rice Genome Annotation Project) [136], TAIR (The *Arabidopsis* Information Resource) [137], the Gene Ontology Database [138], the *Fusarium* Comparative Database [139] and the MIPS *Fusarium graminearum* Genome Database (MIPS) [140]. Generally, a homology was considered as a significant hit according to a threshold at an e-value of ≤1e-20 and a sequence identity of ≥70% in a sequence segment of at least 100 nucleotides for all BLAST analyses [141].

### Quantitative real-time PCR (qPCR) assay

The qPCR expression analyses for selected genes were realised using the 7500 Fast Real-Time System with its corresponding software 7500 v2.0.4 (Applied Biosystems Inc., Foster City, USA). Each reaction contained 5 μl Power SYBR® Green Master Mix (Applied Biosystems Inc., Foster City, USA), 4 ng cDNA, 1 μM of both forward and reverse primer in a final volume of 10 μl. The following thermal profile was used: 2 min at 50°C; 10 min at 94°C; 45 cycles of 45 s at 94°C, 45 s at annealing temperature 60 to 62°C, and 45 s at 72°C. All cDNA samples of each treatment were amplified simultaneously in one PCR plate. After the final PCR cycle, a melting curve analysis was conducted to determine the specificity of the reaction.

Target gene expression was quantified using the comparative 2^-ΔΔCt^ method [142]. The efficiency of each primer pair was determined using 10-fold cDNA dilution series in order to reliable determine the fold changes. The expression of each target gene is presented as fold change normalised to the reference gene ubiquitin (Ta.28553.1.S1_s_at) and relative to the untreated control sample (mock).

Primers for qPCR were designed using the Primer3Plus software [143] based on published EST and gene sequences. Primer sequences together with the used respective EST and gene accession numbers are listed in Additional file 4.

### Chromosomal localisation of the gene *TaMDR1* in wheat

A set of nullisomic-tetrasomic lines (2n = 42) of the spring wheat cultivar Chinese Spring obtained from the Wheat Genetic and Genomic Resources Center, Kansas State University were used to determine the chromosomal location of the *TaMDR1* gene in wheat. Primers designed for qPCR analysis were used for *TaMDR1* gene amplification (Additional file 4).

## Competing interests

'The authors declare that they have no competing interests.

## Authors' contributions

SG carried out the data analysis and interpretation, coordinated the experimental work and drafted the manuscript. BS performed the microarray data analysis and participated in writing the manuscript. SL assisted in the coordination of experimental work, performed qPCR analyses and helped to draft the manuscript. WF supervised the project, contributed to the project design and revised the manuscript. All authors read and approved the final manuscript.

## Supplementary Material

Additional file 1 **Table 1.** Dream FHB-responsive genes categorised as defence related. Supplemental table showing 117 genes that are FHB-responsive induced or repressed in the resistant genotype Dream. Genes were revealed by transcriptome analysis using Affymetrix GeneChip Wheat Genome Array and assigned to 11 gene classes related to a defence response, as well as to the respective timepoints of differential expression. Click here for file

Additional file 2 **Table 2.** Dream genotype-specific genes categorised as defence related. Supplemental table showing 173 constitutive Dream controlled genes. Genes were revealed by transcriptome analysis using Affymetrix GeneChip Wheat Genome Array and assigned to 11 gene classes related to a defence response, as well as to the respective timepoints of differential expression. Click here for file

Additional file 3 **Table 3.** Dream 72 hai-specific genes categorised as defence related. Supplemental table showing 82 genes exclusively differential expressed at the sampling timepoint 72 hai. Genes were revealed by transcriptome analysis using Affymetrix GeneChip Wheat Genome Array and assigned to 11 gene classes related to a defence response, as well as to the respective timepoints of differential expression. Click here for file

Additional file 4 **Table 4.** Sequences of primers used for qPCR analysis of gene expression. Supplemental table showing sequences of primers that were used for the qPCR assays. Accession numbers of Expressed sequence tags (ESTs) and genes that were used to design primers are listed as well. All primers were designed using Primer3Plus software. Click here for file
