# Supplementary material for: Jasmonate and ethylene dependent defence gene expression and suppression of fungal virulence factors: two essential mechanisms of Fusarium head blight resistance in wheat?
Source: BMC Genomics. 2012 Aug 2;13:369. doi: 10.1186/1471-2164-13-369 (PMC3533685; doi:10.1186/1471-2164-13-369)
Supplement: Additional file 1 — Table 1. Dream FHB-responsive genes categorised as defence related. Supplemental table showing 117 genes that are FHB-responsive induced or repressed in the resistant genotype Dream. Genes were revealed by transcriptome analysis using Affymetrix GeneChip Wheat Genome Array and assigned to 11 gene classes related to a defence response, as well as to the respective timepoints of differential expression. [file 1471-2164-13-369-S1.doc]

| **Additional Table 1: FHB-responsive differential up- and down-regulated (+/-) genes (cv. Dream - cv. Lynx 32 and 72 h after *F. graminearum* inoculation); defence-related classes and assignments are based on GSEA analysis and on information obtained from FHB-related literature.** | | | | | | | | | | |
| --- | --- | --- | --- | --- | --- | --- | --- | --- | --- | --- |
| **Gene classes** | **Probe Set** | **Fold change** | | | | | | **Annotation** | |  |
|  |  | **32hai** | | | | **72hai** | |  | |  |
| **JA and ET related genes** |  |  | |  | |  |  |  | |  |
| Lipoxygenases | Ta.1967.2.A1_x_at | 8.19 | | + | |  |  | LOX2.1 (Lipoxygenase 2) | |  |
| Lipoxygenase | TaAffx.104812.1.S1_s_at | 3.66 | | + | |  |  | LOX2.1 (Lipoxygenase 2); chloroplast precursor, putative | |  |
| Lipoxygenases | Ta.13650.1.A1_at | 2.10 | | + | |  |  | LOX6 (Lipoxygenase 6); chloroplastic | |  |
| Jasmonic acid | TaAffx.21290.1.S1_at | 2.13 | | + | |  |  | kelch repeat-containing F-box family protein | |  |
| Ethylene biosynthesis | TaAffx.59867.1.S1_at | 2.45 | | - | |  |  | 1-aminocyclopropane-1-carboxylate oxidase protein, putative | |  |
| Ethylene biosynthesis | Ta.5546.1.S1_a_at | 2.06 | | - | |  |  | 1-aminocyclopropane-1-carboxylate oxidase 2, putative | |  |
| *Fatty acid metabolism and derivatives / Lipid metabolism* | |  | |  | |  |  |  | |  |
| lipid metabolism.TAG synthesis | Ta.30552.1.S1_x_at | 2.50 | | + | | 2.53 | + | moderately similar to diacylglycerol O-acyltransferase | |  |
|  | Ta.22936.1.S1_at | 2.08 | | - | |  |  | lipase, putative | |  |
|  | Ta.7943.1.S1_at | 2.32 | | + | | 2.37 | + | CPA fatty acid synthase | |  |
| lipid metabolic process (GO:0006629) | TaAffx.120727.1.S1_at | 2.61 | | + | |  |  | DUF1295 domain containing protein, putative | |  |
| lipid metabolic process (GO:0006629) | TaAffx.120727.1.S1_x_at | 2.46 | | + | | 2.29 | + | DUF1295 domain containing protein, putative | |  |
| **Cysteine-rich Antimicrobial peptides (AMPs)** | | | | | | | | | | |
| Serine protease inhibitors | Ta.21350.2.S1_at | 2.68 | | + | |  |  | Bowman-Birk type proteinase inhibitor-related protein (wrsi5-1) | PR-06 | |
| Serine protease inhibitors | Ta.2632.2.S1_x_at | 2.25 | | + | |  |  | inhibitor I family protein, putative, expressed | PR-06 | |
| Serine protease inhibitors | Ta.2632.3.S1_x_at | 2.18 | | + | |  |  | inhibitor I family protein, putative, expressed | PR-06 | |
| Non-specific lipid-transfer protein | TaAffx.70203.1.S1_s_at | 2.34 | | + | |  |  | LTP family protein precursor, putative, expressed | PR-14 | |
| Non-specific lipid-transfer protein | Ta.7843.1.S1_a_at | 5.25 | | + | | 10.01 | + | Non-specific lipid-transfer protein 4.3 precursor | PR-14 | |
| Thaumatin | Ta.27762.1.S1_x_at | 2.92 | | - | |  |  | thaumatin, putative, expressed | PR-05 | |
| Thaumatin | Ta.25053.1.S1_at | 2.08 | | - | |  |  | thaumatin, putative, expressed | PR-05 | |
| **Jasmonate-regulated proteins (JRP)** | | | | | | | | | | |
|  | Ta.188.1.S1_at | 21.66 | | + | |  |  | WCI-1; plant disease resistant response; BGAF-like protein | |  |
|  | TaAffx.7388.1.S1_at | 20.87 | | + | |  |  | jacalin-like lectin domain containing protein, putative | |  |
|  | Ta.31.1.S1_at | 3.53 | | + | |  |  | jacalin-like lectin domain containing protein, putative | |  |
| **GDSL-lipases** | | | | | | | | | | |
|  | Ta.13210.1.A1_at | 2.02 | | + | |  |  | GDSL-like lipase/acylhydrolase, putative, expressed | |  |
|  | Ta.21925.1.S1_at | 2.00 | | - | | 2.16 | + | GDSL-like lipase/acylhydrolase, putative, expressed | |  |
| **Proteolysis** | | | | | | | | | | |
|  | TaAffx.134015.1.S1_s_at | 2.43 | | + | | 2.19 | + | OsSCP28 - Putative Serine Carboxypeptidase homologue | |  |
|  | TaAffx.107445.1.S1_s_at | 2.19 | | + | | 2.01 | + | cytosol aminopeptidase family protein | |  |
|  | Ta.4208.1.S1_a_at | 2.11 | | + | |  |  | Putative Serine Carboxypeptidase homologue, expressed | |  |
|  | Ta.9616.3.A1_at | 3.56 | | - | |  |  | OTU-like cysteine protease family protein, putative | |  |
|  | TaAffx.37494.1.A1_at | 2.80 | | - | |  |  | OsSub29 - Putative Subtilisin homologue | |  |
|  | TaAffx.119563.1.S1_at | 2.30 | | - | |  |  | OsSub29 - Putative Subtilisin homologue | |  |
|  | Ta.5333.1.S1_at | 2.07 | | - | | 2.17 | - | ubiquitin family protein, putative | |  |
|  | Ta.14271.2.A1_at | 3.47 | | - | |  |  | CUL4 (CULLIN4); protein binding / ubiquitin-protein ligase | |  |
|  | Ta.9445.1.A1_at | 2.29 | | - | |  |  | AAA family ATPase, putative | |  |
| **Peroxidases** | | | | | | | | | | |
|  | Ta.29496.1.S1_x_at | 2.14 | | - | |  |  | peroxidase precursor, putative | PR-09 | |
| **Genes related to cell wall defense** | | | | | | | | | | |
| *Inactivation of fungal polygalacturonase* | |  | | |  |  |  |  |  | |
|  | Ta.20516.1.S1_at | 8.53 | | + | | 5.46 | + | PGIP1 (polygalacturonase inhibiting protein 1) | PR-02 | |
|  | Ta.20516.1.S1_x_at | 8.40 | | + | | 3.73 | + | PGIP1 (polygalacturonase inhibiting protein 1) | PR-02 | |
| *Inhibition of fungal glycanses* |  |  | |  | |  |  |  |  | |
|  | Ta.29794.1.A1_x_at | 2.56 | | + | | 2.58 | + | xylanase inhibitor |  | |
|  | TaAffx.130061.1.S1_at | 16.41 | | + | | 26.54 | + | pectinesterase inhibitor domain containing protein |  | |
| *Degradation of fungal cell walls* |  |  | |  | |  |  |  |  | |
| GH family 17 / Cellulase family A | TaAffx.131249.1.S1_at | 3.73 | | + | |  |  | glucan endo-1,3-beta-glucosidase precursor, putative, expressed | PR-02 | |
| GH family 17 / Cellulase family A | Ta.20750.1.S1_at | 2.60 | | + | |  |  | glucan endo-1,3-beta-glucosidase precursor, putative, expressed | PR-02 | |
| GH family 17 / Cellulase family A | Ta.22562.1.S1_at | 2.35 | | + | |  |  | glucan endo-1,3-beta-glucosidase precursor, putative, expressed | PR-02 | |
| GH family 5 / Cellulase family A | Ta.4035.1.A1_at | 2.17 | | + | |  |  | glucan endo-1,3-beta-glucosidase precursor, putative, expressed | PR-02 | |
| GH family 16 | Ta.8584.1.S1_at | 4.88 | | - | |  |  | Glucan endo-1,3-beta-D-glucosidase | PR-02 | |
| GH family 16 | Ta.24427.1.S1_at | 2.06 | | - | | 3.79 | - | Glucan endo-1,3-beta-D-glucosidase | PR-02 | |
| GH family 16 | Ta.223.1.S1_at | 2.50 | | - | | 2.99 | - | Glucan endo-1,3-beta-D-glucosidase | PR-02 | |
| GH family 16 | Ta.21297.1.S1_at | 3.48 | | - | | 3.20 | - | Glucan endo-1,3-beta-D-glucosidase | PR-02 | |
| Chitin recognition | Ta.1929.1.S1_at | 2.04 | | - | |  |  | CHIT17 - Chitinase family protein precursor, expressed | PR-02 | |
| **Secondary metabolism and detoxification** | | | | | | | | | | |
| Cytochrome P450s | Ta.8262.1.S1_at | 4.80 | | + | |  |  | cytochrome P450, putative |  | |
| Cytochrome P450s | Ta.19609.1.S1_at | 3.14 | | + | |  |  | CYP72A8 (Cytochrome P72A8) |  | |
| Cytochrome P450s | Ta.21325.1.S1_at | 2.23 | | + | |  |  | cytochrome P450, putative |  | |
| Cytochrome P450s | Ta.1875.2.S1_at | 2.20 | | + | |  |  | cytochrome P450, putative |  | |
| Cytochrome P450s | TaAffx.101303.1.S1_at | 2.19 | | + | |  |  | cytochrome P450 72A1 |  | |
| Cytochrome P450s | Ta.8859.1.S1_at | 2.19 | | + | |  |  | cytochrome P450, putative |  | |
| Cytochrome P450s | Ta.3703.2.S1_at | 2.17 | | + | |  |  | cytochrome P450 72A1 |  | |
| Cytochrome P450s | Ta.22756.1.S1_at | 2.29 | | + | |  |  | cytochrome P450, putative |  | |
| Cytochrome P450s | TaAffx.28047.1.S1_s_at | 3.26 | | - | |  |  | cytochrome P450 51 |  | |
| Cytochrome P450s | TaAffx.81608.1.S1_at | 2.10 | | - | |  |  | cytochrome P450, putative |  | |
| Cytochrome P450s | Ta.6234.1.S1_at | 2.26 | | - | |  |  | cytochrome P450, putative |  | |
| ABC transporter | Ta.4913.2.A1_at | 2.21 | | - | |  |  | ABC-2 type transporter domain containing protein |  | |
| UDP-glycosyltransferase family | Ta.30731.1.S1_at | 2.10 | | + | |  |  | UDP-glucosyl transferase domain containing protein |  | |
| Glutathione S-transferases | Ta.3118.1.S1_at | 2.66 | | + | |  |  | glutathione S-transferase |  | |
| Glutathione S-transferases | Ta.30944.1.S1_at | 2.20 | | - | | 2.36 | - | glutathione S-transferase, putative |  | |
| Flavonoids | Ta.25327.1.A1_at | 2.02 | | + | | 2.49 | + | flavonol synthase (FLS): flavanone 3-hydroxylase |  | |
| Volatile phenylpropanoids | TaAffx.131379.1.A1_at | 3.51 | | - | |  |  | phenylalanine ammonia-lyase, putative |  | |
| Volatile phenylpropanoids | Ta.9220.1.S1_a_at | 2.85 | | - | |  |  | phenylalanine ammonia-lyase, putative |  | |
| Volatile phenylpropanoids | Ta.7022.1.S1_x_at | 2.18 | | - | |  |  | phenylalanine ammonia-lyase, putative |  | |
| Volatile phenylpropanoids | Ta.7022.1.S1_s_at | 2.50 | | - | |  |  | phenylalanine ammonia-lyase, putative |  | |
| Volatile phenylpropanoids | Ta.7022.1.S1_at | 2.06 | | - | |  |  | phenylalanine ammonia-lyase, putative |  | |
| Volatile phenylpropanoids | Ta.7828.1.A1_at | 2.17 | | - | |  |  | moderately similar to ELI3-1 (ELICITOR-ACTIVATED GENE 3) |  | |
| **Miscellaneous defense related genes** | | | | | | | | | | |
|  | Ta.23129.1.S1_x_at | 2.88 | | + | | 2.32 | + | weakly similar to disease resistance-responsive family protein |  | |
|  | Taaffx.23165.2.S1_at | 2.97 | | + | |  |  | weakly similar to leucine-rich repeat family protein |  | |
|  | Ta.97.2.S1_x_at | 2.60 | | - | |  |  | WIR1B PROTEIN |  | |
|  | Ta.22687.1.A1_at | 2.32 | | - | |  |  | disease resistance-responsive protein-related |  | |
|  | Ta.8893.1.S1_at | 2.08 | | - | |  |  | MLO domain containing protein, putative |  | |
|  | Ta.11367.2.S1_at | 2.27 | | - | |  |  | serine/threonine-protein kinase, putative | PR-13 | |
|  | Ta.28655.2.A1_at | 2.35 | | - | |  |  | serine palmitoyltransferase 2, putative | PR-13 | |
| WCI Genes | Ta.21348.2.S1_at | 45.25 | | + | |  |  | WCI-3; sulfur-rich/thionin-like protein | PR-13 | |
| WCI Genes | Ta.21348.1.S1_s_at | 460.38 | | + | | 15.78 | + | WCI-3; sulfur-rich/thionin-like protein | PR-13 | |
| WCI Genes | Ta.21348.1.S1_x_at | 415.87 | | + | | 14.25 | + | WCI-3; sulfur-rich/thionin-like protein | PR-13 | |
| WCI Genes | Ta.191.1.S1_at | 37.62 | | + | | 4.50 | + | WCI-4; thiol protease / cysteine-type endopeptidase activity | PR-13 | |
| WCI Genes | Ta.192.1.S1_at | 2.21 | | - | |  |  | WCI-5; secretory protein |  | |
| **Transcription and signalling** | | | | | | | | | | |
|  | TaAffx.92552.1.S1_at | 2.76 | + | | |  |  | ACR protein: ACT domain containing gene, putative | |  |
|  | TaAffx.50332.1.S1_at | 2.12 | + | | |  |  | NB-ARC domain containing protein | |  |
|  | TaAffx.65732.1.A1_at | 3.48 | - | | |  |  | helix-loop-helix DNA-binding domain containing protein | |  |
|  | TaAffx.629.2.S1_s_at | 2.03 | - | | |  |  | helix-loop-helix DNA-binding domain containing protein | |  |
|  | Ta.23028.1.A1_at | 2.06 | - | | |  |  | basic helix-loop-helix (bHLH) transcription factor, putative | |  |
|  | Ta.25919.1.S1_at | 2.48 | + | | |  |  | myb family transcription factor, putative | |  |
|  | Ta.4125.1.A1_at | 2.16 | + | | | 2.23 | + | myb protein | |  |
|  | Ta.28092.1.S1_s_at | 2.93 | - | | |  |  | myb family transcription factor, putative | |  |
|  | Ta.26049.1.S1_a_at | 2.44 | - | | | 2.14 | + | myb family transcription factor, putative | |  |
|  | TaAffx.106069.1.S1_at | 2.05 | + | | |  |  | zinc finger/CCCH transcription factor, putative | |  |
|  | Ta.6065.1.A1_x_at | 2.41 | + | | |  |  | zinc finger/CCCH transcription factor, putative | |  |
|  | Ta.21196.1.A1_at | 2.11 | + | | |  |  | DNL zinc finger domain containing protein, putative | |  |
|  | Ta.11676.2.S1_at | 2.37 | - | | |  |  | zinc finger, C3HC4 type domain containing protein | |  |
|  | TaAffx.105784.1.S1_at | 2.49 | - | | |  |  | zinc finger helicase family protein, putative | |  |
|  | TaAffx.25368.1.S1_at | 2.01 | + | | |  |  | protein kinase, putative | |  |
|  | TaAffx.128570.1.S1_at | 2.32 | - | | |  |  | protein kinase domain containing protein | |  |
|  | Ta.5029.1.S1_at | 2.29 | - | | | 3.23 | - | protein kinase domain containing protein | |  |
|  | Ta.3640.1.S1_at | 2.04 | - | | | 2.68 | - | protein kinase domain containing protein | |  |
|  | Ta.25379.1.S1_at | 2.04 | - | | |  |  | protein kinase domain containing protein | |  |
|  | Ta.10451.2.A1_x_at | 2.92 | - | | | 2.40 | - | protein kinase domain containing protein | |  |
|  | TaAffx.23192.2.S1_at | 2.10 | - | | | 4.96 | - | resistance-related receptor-like kinase, putative | |  |
|  | TaAffx.82605.1.S1_at | 9.04 | - | | | 11.82 | - | receptor-like protein kinase 2 precursor, putative | |  |
|  | Ta.29629.2.S1_a_at | 3.90 | - | | | 4.34 | - | receptor protein kinase TMK1 precursor, putative | |  |
|  | Ta.23032.2.S1_a_at | 2.04 | + | | |  |  | EF hand family protein, putative | |  |
|  | Ta.5456.1.A1_at | 5.19 | - | | |  |  | WRKY19-b transcription factor | |  |
|  | Ta.25837.1.S1_at | 2.24 | - | | |  |  | OsWRKY10 | |  |
|  | TaAffx.122104.1.S1_s_at | 3.14 | + | | | 2.09 | + | NAC domain transcription factor, putative | |  |
|  | Ta.21150.1.S1_at | 2.19 | + | | | 2.18 | + | transcription initiation factor TFIID subunit 1, putative | |  |
|  | Ta.6053.1.S1_at | 2.47 | - | | | 2.33 | - | putative MAPK protein kinase | |  |
| **Hormone metabolism** | | | | | | | | | | |
| Auxines | Ta.7905.1.S1_a_at | 2.41 | - | | | 2.16 | + | auxin-responsive Aux/IAA gene family member | |  |
| Auxines | TaAffx.128812.1.S1_at | 2.66 | - | | | 2.10 | + | auxin-responsive Aux/IAA gene family member | |  |
| Auxines | Ta.4938.1.S1_at | 2.19 | - | | | 2.17 | + | OsIAA13 - auxin-responsive Aux/IAA gene family member | |  |
| Auxines | Ta.22220.1.S1_at | 2.28 | - | | |  |  | auxin efflux carrier component | |  |
| Auxines | Ta.20938.2.A1_x_at | 2.07 | - | | |  |  | auxin-induced protein 5NG4, putative | |  |
| Gibberellins | Ta.14043.1.S1_at | 3.05 | - | | |  |  | GASR7 - Gibberellin-regulated GASA, expressed | |  |
| Gibberellins | Ta.5616.3.S1_a_at | 2.05 | + | | |  |  | gibberellin receptor GID1L2 | |  |
| Abscisic acid biosythesis | TaAffx.76007.1.S1_at | 2.90 | - | | |  |  | 9-cis-epoxycarotenoid dioxygenase 1, chloroplast precursor | |  |

Only significantly differentially expressed genes (absolute t-value >1.96 and ≥ 2 fold change) are shown in the table.
