# Supplementary material for: Jasmonate and ethylene dependent defence gene expression and suppression of fungal virulence factors: two essential mechanisms of Fusarium head blight resistance in wheat?
Source: BMC Genomics. 2012 Aug 2;13:369. doi: 10.1186/1471-2164-13-369 (PMC3533685; doi:10.1186/1471-2164-13-369)
Supplement: Additional file 2 — Table 2. Dream genotype-specific genes categorised as defence related. Supplemental table showing 173 constitutive Dream controlled genes. Genes were revealed by transcriptome analysis using Affymetrix GeneChip Wheat Genome Array and assigned to 11 gene classes related to a defence response, as well as to the respective timepoints of differential expression. [file 1471-2164-13-369-S2.doc]

| **Additional Table 2: Dream genotype-specific differential up- and down-regulated (+/-) genes (cv. Dream - cv. Lynx 32 h and 72h after *F. graminearum* and mock inoculation); defence-related classes and assignments are based on GSEA analysis and on information obtained from FHB-related literature** | | | | | | | | | | | |
| --- | --- | --- | --- | --- | --- | --- | --- | --- | --- | --- | --- |
| **Gene classes** | **Probe Set** | **Fold change** | | | | | | | **Annotation** | |  |
|  |  | **32 hai** | | | | **72 hai** | | |  | |  |
| **JA and ET related genes** | | | | | | | | | | | |
| Lipoxygenase | Ta.1967.1.S1_x_at | 11.18 | + |  | | | |  | LOX2.1 (Lipoxygenase 2) | |  |
| Lipoxygenase | Ta.485.1.A1_at | 6.45 | + | 3.68 | | | | + | LOX 2.3 (Lipoxygenase 2.3) | |  |
| Lipoxygenase | Ta.22828.2.S1_at | 3.22 | + |  | | | |  | LOX2 (Lipoxygenase 2) | |  |
| Lipoxygenase | TaAffx.90316.1.S1_at | 20.39 | + | 14.96 | | | | + | lipoxygenase, putative | |  |
| Lipoxygenase | Ta.23763.1.S1_at | 55.84 | + | 51.63 | | | | + | WCI-2; lipoxygenase, putative | |  |
| Jasmonic acid | Ta.8836.1.A1_at | 3.56 | + |  | | | |  | kelch repeat-containing F-box family protein | |  |
| Ethylene biosynthesis | Ta.22475.1.S1_at | 6.60 | + | 8.48 | | | | + | 1-aminocyclopropane-1-carboxylate oxidase, putative | |  |
| Ethylene biosynthesis | Ta.11292.1.A1_at | 3,41 | + | 6,77 | | | | + | 1-aminocyclopropane-1-carboxylate oxidase homolog 2, putative | |  |
| Ethylene biosynthesis | TaAffx.72199.1.A1_at | 2.78 | + | 8.88 | | | | + | 1-aminocyclopropane-1-carboxylate oxidase homolog 1 | |  |
| Ethylene biosynthesis | Ta.425.2.S1_at | 3.36 | - | 3.83 | | | | - | 1-aminocyclopropane-1-carboxylate oxidase | |  |
| Ethylene biosynthesis | Ta.9107.2.S1_at | 5.70 | - | 3.82 | | | | - | 1-aminocyclopropane-1-carboxylate oxidase protein, putative | |  |
| Ethylene biosynthesis | Ta.9107.2.S1_a_at | 3.94 | - | 2.36 | | | | - | 1-aminocyclopropane-1-carboxylate oxidase protein, putative | |  |
| Ethylene biosynthesis | Ta.9107.1.S1_x_at | 3.56 | - | 2.43 | | | | - | 1-aminocyclopropane-1-carboxylate oxidase protein, putative | |  |
| Ethylene biosynthesis | TaAffx.72199.2.S1_at | 2.24 | - |  | | | |  | 1-aminocyclopropane-1-carboxylate oxidase homolog 1, putative | | |
| *Fatty acid metabolism and derivatives / Lipid metabolism* | | | | | | | | | | | |
|  | Ta.556.1.S1_at | 3.89 | - |  | | | |  | lipase, putative | |  |
|  | Ta.556.1.S1_x_at | 4.42 | - |  | | | |  | lipase, putative | |  |
| **Cysteine-rich Antimicrobial peptides (AMPs)** | | | | | | | | | | | |
| Serine protease inhibitors | Ta.30711.1.S1_x_at | 2.45 | + |  | | | |  | Bowman-Birk type proteinase inhibitor-related protein (wrsi5-1) | | PR-06 |
| Non-specific lipid-transfer protein | Ta.19064.1.S1_x_at | 25.81 | + | 10.13 | | | | + | LTP family protein precursor | | PR-14 |
| Non-specific lipid-transfer protein | Ta.21646.1.S1_x_at | 46.42 | + | 67.03 | | | | + | LTP family protein precursor | | PR-14 |
| Non-specific lipid-transfer protein | Ta.21646.1.S1_at | 45.89 | + | 47.07 | | | | + | LTP family protein precursor | | PR-14 |
| Non-specific lipid-transfer protein | Ta.23917.3.S1_x_at | 3.02 | - |  | | | |  | LTP family protein precursor | | PR-14 |
| Thionin | Ta.23967.1.S1_s_at | 8.57 | + | 5.76 | | | | + | THION1.2 - Plant thionin family protein precursor, putative | | PR-13 |
| Thionin | Ta.23967.1.S1_x_at | 4.44 | + |  | | | |  | THION1 - Plant thionin family protein precursor, putative | | PR-13 |
| Defensin | Ta.20930.1.S1_at | 20.58 | + | 3.00 | | | | + | Defensin precursor | | PR-12 |
| Defensin | Ta.28319.1.S1_at | 13.99 | + |  | | | |  | Defensin and Defensin-like DEFL family | | PR-12 |
| **GDSL-lipases** | | | | | | | | | | | |
|  | Ta.9671.3.S1_x_at | 3.76 | + |  | | | |  | GDSL-like lipase/acylhydrolase, putative, expressed | |  |
|  | TaAffx.2026.1.S1_at | 3.37 | + |  | | | |  | GDSL-like lipase/acylhydrolase, putative, expressed | |  |
|  | TaAffx.55993.1.S1_at | 2.31 | + |  | | | |  | GDSL-like lipase/acylhydrolase, putative, expressed | |  |
|  | Ta.9671.2.S1_x_at | 57.95 | + | 32.00 | | | | + | GDSL-like lipase/acylhydrolase, putative, expressed | |  |
|  | Ta.9671.3.S1_a_at | 57.02 | + | 13.18 | | | | + | GDSL-like lipase/acylhydrolase, putative, expressed | |  |
|  | Ta.9671.2.S1_at | 50.10 | + | 26.85 | | | | + | GDSL-like lipase/acylhydrolase, putative, expressed | |  |
|  | Ta.9671.1.S1_x_at | 32.98 | + | 4.13 | | | | + | GDSL-like lipase/acylhydrolase, putative, expressed | |  |
|  | Ta.25390.2.S1_at | 17.39 | + | 13.86 | | | | + | GDSL-like lipase/acylhydrolase, putative, expressed | |  |
|  | Ta.25390.2.S1_x_at | 16.76 | + | 11.55 | | | | + | GDSL-like lipase/acylhydrolase, putative, expressed | |  |
|  | TaAffx.106377.1.S1_at | 2.51 | + | 2.69 | | | | + | GDSL-like lipase/acylhydrolase, putative, expressed | |  |
|  | Ta.9046.2.S1_a_at | 3.32 | - |  | | | |  | GDSL-like lipase/acylhydrolase, putative, expressed | |  |
|  | Ta.9046.1.S1_at | 3.20 | - |  | | | |  | GDSL-like lipase/acylhydrolase, putative, expressed | |  |
|  | Ta.8094.2.S1_at | 6.13 | - | 4.38 | | | | - | GDSL-like lipase/acylhydrolase, putative, expressed | |  |
|  | Ta.10106.1.S1_at | 2.32 | - | 3.17 | | | | - | GDSL-like lipase/acylhydrolase, putative, expressed | |  |
| **Proteolysis** | | | | | | | | | | |  |
|  | TaAffx.119683.1.A1_at | 45,99 | + | 17,88 | | | | + | putative serine carboxypeptidase homologue, expressed | |  |
|  | Ta.7124.1.A1_at | 41,64 | + | 41,07 | | | | + | serine protease-like protein | |  |
|  | TaAffx.28669.1.A1_at | 7,62 | + | 11,71 | | | | + | putative serine carboxypeptidase homologue | |  |
|  | TaAffx.92608.1.S1_at | 2,54 | + |  | | | |  | putative serine carboxypeptidase homologue, expressed | |  |
|  | Ta.6399.3.S1_at | 3,39 | + | 4,95 | | | | + | serine protease-like protein | |  |
|  | Ta.26095.1.S1_at | 4,53 | + | 4,12 | | | | + | cytosol aminopeptidase | |  |
|  | Ta.408.2.S1_at | 2,49 | + | 9,08 | | | | + | subtilase family protein | |  |
|  | TaAffx.93139.1.A1_at | 26,11 | + | 20,68 | | | | + | BB (BIG BROTHER); protein binding / ubiquitin-protein ligase | |  |
|  | Ta.14571.2.A1_at | 15,93 | + | 15,89 | | | | + | EMB2107 (EMBRYO DEFECTIVE 2107) | |  |
|  | TaAffx.119984.1.S1_at | 2,24 | + |  | | | |  | +L5 (UBIQUITIN PROTEIN LIGASE 5); acid-amino acid ligase | |  |
|  | Ta.408.3.S1_at | 2,47 | + | 7,40 | | | | + | subtilase family protein | |  |
|  | Ta.408.1.A1_at | 2,46 | + | 7,21 | | | | + | subtilase family protein | |  |
|  | TaAffx.91995.1.A1_at | 3,48 | + | 5,29 | | | | + | OsPOP9 - Putative Prolyl Oligopeptidase homologue | |  |
|  | Ta.28553.4.S1_x_at | 4,56 | - | 3,81 | | | | - | ubiquitin family protein, putative | |  |
|  | Ta.14571.1.S1_at | 73,69 | - | 60,27 | | | | - | proteasome subunit, putative | |  |
|  | Ta.18187.1.S1_at | 3,44 | - | 2,31 | | | | - | peptidase, M24 family protein, putative | |  |
|  | Ta.3501.2.S1_x_at | 2,01 | - | 2,20 | | | | - | OsFtsH5 FtsH protease, homologue of AtFtsH4 | |  |
|  | Ta.27445.1.S1_at | 122,79 | - | 180,60 | | | | - | OsFtsH1 FtsH protease, homologue of AtFtsH1/5 | |  |
|  |  |  |  |  | | | |  |  | |  |
| **Peroxidases** | | | | | | | | | | | |
|  | Ta.12472.1.S1_x_at | 4.12 | + |  | | | |  | peroxidase precursor, putative | | PR-09 |
|  | Ta.29603.1.A1_at | 3.15 | + |  | | | |  | peroxidase, putative | | PR-09 |
|  | Ta.17325.1.S1_x_at | 8.86 | + | 10.60 | | | | + | peroxidase, putative | | PR-09 |
|  | Ta.17325.1.S1_at | 8.77 | + | 11.71 | | | | + | peroxidase, putative | | PR-09 |
|  | Ta.25333.1.S1_at | 4.22 | - |  | | | |  | peroxidase precursor, putative | | PR-09 |
|  | Ta.2746.2.S1_x_at | 3,45 | - |  | | | |  | peroxidase precursor, putative | | PR-09 |
|  | Ta.2746.1.S1_a_at | 3,08 | - |  | | | |  | peroxidase precursor, putative | | PR-09 |
|  | Ta.2746.1.S1_x_at | 2,89 | - |  | | | |  | peroxidase precursor, putative | | PR-09 |
|  | Ta.30698.1.S1_at | 2,71 | - |  | | | |  | peroxidase precursor, putative | | PR-09 |
|  | Ta.23376.2.S1_s_at | 2,69 | - |  | | | |  | peroxidase precursor, putative | | PR-09 |
| **Genes related to cell wall defense** | | | | | | | | | | | |
| *Inactivation of fungal polygalacturonase* | | | | | | | | | | | |
|  | TaAffx.11210.1.S1_x_at | 3,90 | + | 3,08 | | | | + | glycoside hydrolase family 28 protein / polygalacturonase (pectinase) | | |
| *Inhibition of fungal glycanses* | | | | | | | | | | | |
|  | Ta.1200.1.S1_x_at | 4,89 | + | | 4,16 | | + | | xylanase inhibitor protein I |  | |
|  | Ta.1200.1.S1_at | 4,68 | + | | 4,26 | | + | | xylanase inhibitor protein I |  | |
| *Degradation of fungal cell walls* | | | | | | | | | | | |
| GH family 17 / Cellulase family A | Ta.21069.1.S1_a_at | 25,52 | + | 4,08 | | | | + | glucan 1,3-beta-glucosidase, putative | | PR-02 |
| GH family 17 / Cellulase family A | Ta.21069.3.S1_x_at | 8,04 | + | 2,95 | | | | + | glucan 1,3-beta-glucosidase, putative | | PR-02 |
| GH family 17 / Cellulase family A | Ta.20421.1.S1_at | 5,00 | + | 8,46 | | | | + | glucan 1,3-beta-glucosidase, putative | | PR-02 |
|  | Ta.4683.1.S1_at | 3,87 | - |  | | | |  | beta-glucosidase, exo-beta-glucanse | |  |
|  | TaAffx.119729.1.S1_at | 2,47 | - |  | | | |  | beta-glucosidase homologue | |  |
|  | Ta.4191.1.A1_at | 2,10 | - |  | | | |  | beta-glucosidase homologue | |  |
|  | TaAffx.101246.1.S1_at | 2,31 | + | 2,42 | | | | + | beta-glucosidase | |  |
| **Secondary metabolism and detoxification** | | | | | | | | | | |  |
| Cytochrome P450s | Ta.9114.2.S1_at | 6,20 | + | 2,63 | | | | + | cytochrome P450, putative | |  |
| Cytochrome P450s | Ta.1875.1.S1_at | 2,21 | + |  | | | |  | cytochrome P450, putative | |  |
| Cytochrome P450s | Ta.174.1.S1_at | 9,04 | - | 11,82 | | | | - | cytochrome P450, putative | |  |
| Cytochrome P450s | TaAffx.12524.1.A1_at | 7,18 | - | 26,60 | | | | - | cytochrome P450, putative | |  |
| Cytochrome P450s | Ta.9332.2.A1_x_at | 4,49 | - | 2,43 | | | | - | cytochrome P450, putative | |  |
| Cytochrome P450s | Ta.9332.2.A1_at | 3,94 | - | 2,37 | | | | - | cytochrome P450, putative | |  |
| Cytochrome P450s | TaAffx.12442.1.A1_at | 3,78 | - | 3,11 | | | | - | cytochrome P450, putative | |  |
| Cytochrome P450s | Ta.9332.1.S1_x_at | 2,33 | - | 2,54 | | | | - | cytochrome P450, putative | |  |
| Cytochrome P450s | TaAffx.93492.1.S1_at | 4,67 | - | 6,08 | | | | - | cytochrome P450, putative | |  |
| Cytochrome P450s | Ta.5479.1.A1_x_at | 4,33 | - | 4,88 | | | | - | cytochrome P450, putative | |  |
| Cytochrome P450s | TaAffx.31416.1.S1_at | 23,05 | - | 10,58 | | | | - | cytochrome P450 72A1, putative | |  |
| Cytochrome P450s | TaAffx.28047.1.S1_at | 6,38 | - |  | | | |  | cytochrome P450 51, putative | |  |
|  | Ta.25693.1.A1_at | 2,09 | + | 2,61 | | | | + | MATE efflux family protein | |  |
|  | Ta.8460.1.S1_at | 3,48 | + | 2,68 | | | | + | MATE efflux family protein | |  |
|  | TaAffx.117641.1.S1_at | 2,63 | + | 3,10 | | | | + | MATE efflux family protein | |  |
|  | Ta.13572.1.S1_at | 7,46 | + | 5,97 | | | | + | ABC-type phosphate transport system | |  |
|  | Ta.5888.1.S1_s_at | 2,18 | + | 18,25 | | | | + | polar amino acid ABC transporter | |  |
|  | TaAffx.31520.1.S1_at | 6,50 | + | 3,32 | | | | + | 2-oxoglutarate-dependent dioxygenase | |  |
|  | Ta.12050.1.A1_at | 2,76 | + |  | | | |  | 2-oxoglutarate-dependent dioxygenase | |  |
|  | Ta.932.1.S1_at | 2,20 | + |  | | | |  | zinc-binding alcohol dehydrogenase domain-containing protein 2 | | |
| UDP-glycosyltransferase family | TaAffx.410.1.S1_at | 36,09 | + | 19,12 | | | | + | UDP-glycosyltransferase | |  |
| UDP-glycosyltransferase family | Ta.1450.3.S1_at | 2,79 | + | 7,60 | | | | + | UDP-D-glucose epimerase 1 | |  |
| UDP-glycosyltransferase family | Ta.12214.1.A1_at | 32,52 | + | 81,38 | | | | + | UDP-glucose glucosyltransferase | |  |
| UDP-glycosyltransferase family | Ta.10376.1.A1_at | 2,35 | + | 2,73 | | | | + | glycosyltransferase | |  |
| UDP-glycosyltransferase family | TaAffx.49246.1.A1_at | 3,47 | + | 4,18 | | | | + | cytokinin-O-glucosyltransferase 2, putative | |  |
| UDP-glycosyltransferase family | TaAffx.92142.1.S1_at | 2,38 | - |  | | | |  | glycosyltransferase, putative | |  |
| UDP-glycosyltransferase family | TaAffx.42526.1.S1_at | 3,39 | - |  | | | |  | glycosyl transferase 8 domain containing protein, putative | |  |
| UDP-glycosyltransferase family | Ta.9000.1.S1_at | 2,58 | - |  | | | |  | glycosyl hydrolases, putative | |  |
| UDP-glycosyltransferase family | TaAffx.111940.1.S1_x_at | 2,36 | - |  | | | |  | glycosyl hydrolases, putative | |  |
| UDP-glycosyltransferase family | Ta.30703.1.S1_at | 4,22 | - | 5,02 | | | | - | glycosyl hydrolases family 16, putative | |  |
| UDP-glycosyltransferase family | TaAffx.15847.2.S1_at | 2,27 | - | 2,36 | | | | - | glycosyl hydrolases family 16, putative | |  |
| UDP-glycosyltransferase family | Ta.4179.1.S1_at | 2,74 | - |  | | | |  | glycosyl hydrolase family protein 27, putative | |  |
| Glutathione S-transferases | Ta.14856.1.S1_at | 2,19 | + |  | | | |  | GSH2 (glutathione synthetase 2) | |  |
| Glutathione S-transferases | Ta.24150.1.S1_at | 134,05 | + | 310,83 | | | | + | glutathione-S-transferase | |  |
| Glutathione S-transferases | Ta.16787.1.A1_at | 7,00 | + | 4,77 | | | | + | glutathione S-transferase | |  |
| Glutathione S-transferases | TaAffx.64719.1.A1_at | 4,22 | + | 3,14 | | | | + | glutathione S-transferase | |  |
| Favonoids | Ta.27553.1.S1_at | 3,11 | + |  | | | |  | chalcone synthase, putative | |  |
| Favonoids | Ta.28700.1.S1_at | 4,72 | - |  | | | |  | chalcone synthase, putative | |  |
| Favonoids | TaAffx.15439.1.A1_at | 10,17 | - |  | | | |  | chalcone and stilbene synthases, putative | |  |
| Favonoids | Ta.7223.3.S1_x_at | 3,70 | - | 2,57 | | | | - | flavonol synthase/flavanone 3-hydroxylase, putative | |  |
| **Miscellaneous defense related genes** | | | | | | | | | | | |
| PPIase_Cyclophilin-type | Ta.9153.3.S1_a_at | 3,21 | + | 2,76 | | | | + | ROC4, peptidyl-prolyl cis-trans isomerase, chloroplast / cyclophilin | | |
| PPIase_Cyclophilin-type | Ta.9153.1.S1_at | 12,58 | + | 9,51 | | | | + | ROC4, peptidyl-prolyl cis-trans isomerase, chloroplast / cyclophilin | | |
| PPIase_Cyclophilin-type | Ta.13731.1.S1_at | 8,06 | + | 10,88 | | | | + | peptidyl-prolyl cis-trans isomerase activity, isomerase activity | |  |
|  | Ta.23129.1.S1_at | 3,16 | + | 2,43 | | | | + | disease resistance-responsive family protein | |  |
|  | Ta.24087.2.S1_a_at | 2,55 | - | 2,62 | | | | - | RGH1A (Mla locus), putative | |  |
|  | TaAffx.42397.1.S1_at | 7,89 | - | 8,13 | | | | - | resistance-related receptor-like kinase, putative | |  |
|  | Ta.27275.1.S1_at | 6,44 | - | 3,56 | | | | - | resistance protein, putative | |  |
|  | TaAffx.104854.1.S1_at | 2,82 | - |  | | | |  | resistance protein, putative | |  |
|  | Ta.2931.1.A1_at | 4,62 | - | 4,94 | | | | - | disease resistance protein RPM1, putative | |  |
|  | Ta.24195.2.S1_at | 5,86 | - | 6,12 | | | | - | disease resistance protein RGA3, putative | |  |
|  | TaAffx.28806.1.S1_at | 2,74 | - |  | | | |  | disease resistance protein RGA3, putative | |  |
|  | TaAffx.119753.1.S1_at | 4,15 | - |  | | | |  | NBS-LRR disease resistance protein, putative | |  |
| **Transcription and signalling** | | | | | | | | | | | |
|  | Ta.12834.1.S1_s_at | 2,62 | + | |  | |  | | myb59 (myb domain protein 59) |  | |
|  | Ta.27013.1.S1_at | 2,54 | + | |  | |  | | myb family transcription factor |  | |
|  | Ta.4965.3.S1_at | 7,82 | + | | 5,92 | | + | | myb family transcription factor |  | |
|  | Ta.19783.1.S1_x_at | 4,86 | + | | 2,68 | | + | | myb111 (myb domain protein 111) |  | |
|  | Ta.3031.1.A1_at | 2,53 | - | |  | |  | | MYB family transcription factor, putative |  | |
|  | TaAffx.113352.2.S1_at | 2,07 | + | |  | |  | | bZIP transcription factor domain containing protein |  | |
|  | Ta.24806.2.S1_at | 8,00 | + | | 4,18 | | + | | bZIP transcription factor |  | |
|  | Ta.13724.1.S1_at | 2,31 | - | | 2,49 | | - | | bZIP transcription factor domain containing protein |  | |
|  | Ta.30908.1.S1_at | 88,03 | + | | 99,04 | | + | | EF hand family protein, putative |  | |
|  | TaAffx.110751.1.S1_s_at | 8,02 | + | | 5,31 | | + | | EF hand family protein, putative, expressed |  | |
|  | Ta.22681.1.S1_at | 5,78 | - | | 11,93 | | - | | EF hand family protein, putative |  | |
|  | Ta.7853.1.S1_at | 8,73 | + | | 7,84 | | + | | zinc finger (FYVE type) family protein |  | |
|  | TaAffx.9420.1.S1_s_at | 2,39 | + | | 2,80 | | + | | zinc finger (C3HC4-type RING finger) family protein |  | |
|  | Ta.23352.1.S1_at | 112,73 | + | | 138,78 | | + | | protein kinase domain containing protein |  | |
|  | Ta.26080.2.A1_at | 80,82 | + | | 71,67 | | + | | protein kinase, putative |  | |
|  | Ta.26080.1.A1_s_at | 22,94 | + | | 17,79 | | + | | protein kinase, putative |  | |
|  | Ta.12647.2.S1_at | 8,30 | + | | 6,70 | | + | | protein kinase-like protein |  | |
|  | Ta.26080.1.A1_a_at | 7,96 | + | | 5,60 | | + | | protein kinase, putative |  | |
|  | Ta.8986.1.A1_at | 2,09 | + | | 2,03 | | + | | putative serine/threonine-protein kinase related cluster |  | |
|  | TaAffx.11860.2.S1_at | 2,08 | + | | 2,06 | | + | | protein kinase domain containing protein |  | |
|  | TaAffx.94232.1.S1_at | 3,39 | + | | 2,65 | | + | | receptor-like kinase, putative |  | |
|  | Ta.29629.1.S1_s_at | 3,57 | - | | 3,06 | | - | | receptor protein kinase TMK1 precursor, putative |  | |
|  | Ta.6407.1.A1_at | 2,23 | - | | 3,18 | | - | | protein kinase, putative |  | |
|  | TaAffx.4849.1.S1_at | 4,49 | - | | 2,45 | | - | | protein kinase domain containing protein |  | |
| Cysteine-rich receptor-like kinases | TaAffx.27775.1.S1_at | 95,23 | + | | 61,11 | | + | | DUF26 kinase_cysteine-rich receptor-like protein kinase |  | |
| Cysteine-rich receptor-like kinases | TaAffx.105530.1.S1_at | 153,63 | + | | 30,98 | | + | | DUF26 kinase_cysteine-rich receptor-like protein kinase |  | |
| Cysteine-rich receptor-like kinases | Ta.5331.1.A1_x_at | 5,75 | - | | 5,64 | | - | | cys-rich receptor-like protein kinase 21 precursor, putative |  | |
| Cysteine-rich receptor-like kinases | Ta.5331.1.A1_a_at | 4,55 | - | | 3,60 | | - | | cys-rich receptor-like protein kinase 21 precursor, putative |  | |
| Cysteine-rich receptor-like kinases | Ta.8165.2.S1_a_at | 2,91 | - | | 2,85 | | - | | cys-rich receptor-like protein kinase 21 precursor, putative |  | |
| Cysteine-rich receptor-like kinases | Ta.941.1.A1_at | 4,03 | - | | 4,34 | | - | | cys-rich receptor-like protein kinase 21 precursor, putative |  | |
| LRR-proteins | Ta.6945.1.A1_a_at | 3,62 | - | |  | |  | | leucine-rich repeat family protein, putative |  | |
| LRR-proteins | Ta.6945.2.S1_at | 2,35 | - | |  | |  | | leucine-rich repeat family protein, putative |  | |
| LRR-proteins | Ta.6945.1.A1_at | 2,16 | - | |  | |  | | leucine-rich repeat family protein, putative |  | |
| LRR-proteins | Ta.25825.1.A1_at | 5,51 | - | | 3,07 | | - | | lectin protein kinase family protein, putative |  | |
|  | TaAffx.82723.1.A1_at | 7,96 | - | | 6,81 | | - | | cadmium tolerance factor, putative |  | |
|  | Ta.8614.1.S1_at | 2,05 | - | |  | |  | | OsWRKY45 |  | |
|  | Ta.26917.1.S1_at | 2,59 | - | | 2,68 | | - | | OsMADS65 - MADS-box family gene (MIKC type) |  | |
|  | Ta.7594.2.S1_at | 2,32 | - | |  | |  | | OsMADS34 - MADS-box family gene (MIKC type) |  | |
|  | Ta.2890.1.S1_at | 2,15 | - | |  | |  | | OsMADS1 - MADS-box family gene (MIKC type) |  | |
|  | Ta.25981.1.A1_at | 10,80 | - | | 4,97 | | - | | NB-ARC domain containing protein |  | |
|  | TaAffx.12459.1.S1_at | 4,26 | - | | 6,05 | | - | | NB-ARC domain containing protein |  | |
| **Hormone metabolism** | | | | | | | | | | | |
| Auxines | TaAffx.37740.1.S1_s_at | 3,47 | - | 3,59 | | | | - | auxin efflux carrier component, putative, expressed | |  |
| Auxines | Ta.21285.1.A1_s_at | 2,70 | - | 2,72 | | | | - | auxin-responsive SAUR gene family member | |  |
| Auxines | Ta.1600.1.A1_at | 5,05 | - |  | | | |  | auxin-induced protein 5NG4, putative | |  |

Only significantly differentially expressed genes (absolute t-value >1.96 and ≥ 2 fold change) are shown in the table.
