# Supplementary material for: Jasmonate and ethylene dependent defence gene expression and suppression of fungal virulence factors: two essential mechanisms of Fusarium head blight resistance in wheat?
Source: BMC Genomics. 2012 Aug 2;13:369. doi: 10.1186/1471-2164-13-369 (PMC3533685; doi:10.1186/1471-2164-13-369)
Supplement: Additional file 3 — Table 3. Dream 72 hai-specific genes categorised as defence related. Supplemental table showing 82 genes exclusively differential expressed at the sampling timepoint 72 hai. Genes were revealed by transcriptome analysis using Affymetrix GeneChip Wheat Genome Array and assigned to 11 gene classes related to a defence response, as well as to the respective timepoints of differential expression. [file 1471-2164-13-369-S3.doc]

| **Additional Table 3: 72 hai-specific differential up- and down-regulated (+/-) genes (cv. Dream - cv. Lynx 72 hai after *F. graminearum* inoculation); defence-related classes and assignments are based on GSEA analysis and on information obtained from FHB-related literature.** | | | | | | | |
| --- | --- | --- | --- | --- | --- | --- | --- |
| **Gene classes** | **Probe Set** | **Fold change** | | | | **Annotation** |  |
|  |  | **32 hai** | | **72 hai** | |  |  |
| **JA and ET related genes** | |  |  |  |  |  |  |
| Jasmonic acid | TaAffx.108190.1.S1_at |  |  | 2,22 | + | kelch repeat-containing F-box family protein |  |
| Jasmonic acid | Ta.30921.2.S1_at |  |  | 2,17 | + | OPR2 (12-oxophytodienoate reductase 2) |  |
| Jasmonic acid | Ta.6684.1.S1_at |  |  | 2,23 | + | acyltransferase, putative |  |
| *Fatty acid metabolism and derivatives / Lipid metabolism* | |  |  |  |  |  |  |
|  | Ta.27086.1.S1_at |  |  | 2,23 | + | lipase precursor, putative |  |
|  | TaAffx.86362.1.S1_at |  |  | 2,25 | + | lipase precursor, putative |  |
|  |  |  |  |  |  |  |  |
| **Cysteine-rich Antimicrobial peptides (AMPs)** | |  |  |  |  |  |  |
| Serine protease inhibitors | Ta.22614.1.S1_at |  |  | 9,49 | + | inhibitor I family protein, putative, expressed | PR-06 |
| Non-specific lipid-transfer protein | Ta.30504.1.A1_at |  |  | 3,15 | + | LTP family protein precursor, expressed | PR-14 |
| Non-specific lipid-transfer protein | Ta.18647.3.S1_at |  |  | 2,09 | + | LTP family protein precursor, expressed | PR-14 |
| **GDSL-lipases** |  |  |  |  |  |  |  |
|  | TaAffx.97535.1.S1_at |  |  | 2,18 | + | GDSL-like lipase/acylhydrolase, putative, expressed |  |
| **Proteolysis** | | | | | | | |
|  | TaAffx.32049.1.S1_at |  |  | 4,66 | + | OsSub35 - Putative Subtilisin homologue |  |
|  | TaAffx.64664.1.S1_at |  |  | 3,02 | + | prolyl oligopeptidase, putative |  |
|  | TaAffx.121307.1.S1_at |  |  | 3,92 | + | putative serine carboxypeptidase homologue |  |
|  | TaAffx.134015.1.S1_x_at |  |  | 2,32 | + | putative serine carboxypeptidase homologue |  |
|  | Ta.28003.1.S1_x_at |  |  | 2,25 | - | aspartyl protease family protein |  |
|  | Ta.16231.1.A1_at |  |  | 2,26 | - | UBIQUITIN-CONJUGATING ENZYME 26 |  |
|  | Ta.14475.1.S1_at |  |  | 2,83 | - | EARLY-RESPONSIVE TO DEHYDRATION 16 |  |
| **Peroxidases** | |  |  |  |  |  |  |
|  | TaAffx.16965.1.S1_at |  |  | 3,65 | + | peroxidase precursor, putative | PR-09 |
|  | Ta.25918.1.A1_at |  |  | 2,25 | + | peroxidase precursor, putative | PR-09 |
|  | Ta.9420.1.S1_at |  |  | 2,22 | + | peroxidase precursor, putative | PR-09 |
|  | Ta.13307.1.S1_x_at |  |  | 2,24 | + | peroxidase precursor, putative | PR-09 |
|  | TaAffx.97820.1.S1_at |  |  | 2,77 | - | peroxidase precursor, putative | PR-09 |
|  | Ta.24715.1.S1_at |  |  | 9,08 | - | peroxidase, putative | PR-09 |
| **Genes related to cell wall defense** | |  |  |  |  |  |  |
| *Inhibition of fungal glycanses* |  |  |  |  |  |  |  |
|  | Ta.29434.1.A1_at |  |  | 2,32 | + | pectinesterase inhibitor domain containing protein, putative, expressed |  |
| *Degradation of fungal cell walls* |  |  |  |  |  |  |  |
|  | Ta.5592.1.S1_at |  |  | 3,11 | - | chitinase family protein precursor | PR-02 |
| **Secondary metabolism and detoxification** | |  |  |  |  |  |  |
| Cytochrome P450s | TaAffx.42466.1.S1_at |  |  | 5,59 | + | cytochrome P450, putative |  |
| Cytochrome P450s | Ta.11147.1.A1_at |  |  | 2,42 | + | cytochrome P450, putative |  |
| Cytochrome P450s | Ta.11257.1.A1_at |  |  | 2,19 | + | cytochrome P450, putative |  |
| Cytochrome P450s | TaAffx.37978.1.A1_at |  |  | 2,98 | + | cytochrome P450, putative |  |
| Cytochrome P450s | TaAffx.64667.1.S1_at |  |  | 2,22 | + | cytochrome P450, putative |  |
| Cytochrome P450s | TaAffx.118836.1.S1_at |  |  | 2,09 | + | cytochrome P450, putative |  |
|  | Ta.12133.1.A1_at |  |  | 2,05 | + | MATE efflux family protein, putative |  |
|  | Ta.25708.1.A1_at |  |  | 2,07 | + | MATE efflux protein, putative |  |
| UDP-glycosyltransferase family | TaAffx.30022.1.S1_at |  |  | 2,30 | + | UDP-Gal-lipooligosaccharide galactosyltransferase |  |
| UDP-glycosyltransferase family | Ta.3596.1.S1_x_at |  |  | 2,16 | + | UDP-glucose 6-dehydrogenase, putative |  |
| UDP-glycosyltransferase family | Ta.30327.2.A1_at |  |  | 2,21 | + | anthocyanidin 5,3-O-glucosyltransferase |  |
| UDP-glycosyltransferase family | TaAffx.17365.2.A1_at |  |  | 2,51 | + | glycosyl transferase 8 domain containing protein, putative |  |
| UDP-glycosyltransferase family | Ta.5628.1.A1_at |  |  | 2,34 | + | glycosyltransferase family 43 protein, putative |  |
| UDP-glycosyltransferase family | Ta.22376.1.S1_at |  |  | 2,03 | + | glycosyltransferase, putative |  |
| Glutathione S-transferases | Ta.1019.2.A1_a_at |  |  | 2,74 | + | glutathione S-transferase domain |  |
| Glutathione S-transferases | TaAffx.78483.1.S1_at |  |  | 2,04 | + | glutathione S-transferase |  |
| Glutathione S-transferases | Ta.2397.1.S1_at |  |  | 2,01 | + | glutathione S-transferase, N-terminal domain containing protein |  |
| Glutathione S-transferases | Ta.237.1.S1_at |  |  | 2,01 | - | ATGSTZ1 (Glutathione S-transferase 18) |  |
| Glutathione S-transferases | TaAffx.110629.1.S1_at |  |  | 2,16 | - | ATGSTU7 (Glutathione S-transferase 18) |  |
| Glutathione S-transferases | TaAffx.110629.1.S1_x_at |  |  | 2,17 | - | ATGSTU7 (Glutathione S-transferase 18) |  |
| Volatile phenylpropanoids | Ta.20429.1.S1_at |  |  | 2,18 | + | phenylalanine ammonia-lyase |  |
| Volatile phenylpropanoids | TaAffx.57491.1.S1_x_at |  |  | 2,08 | + | phenylalanine ammonia-lyase, putative |  |
| Isoflavonoid biosynthesis | Ta.9172.1.S1_at |  |  | 4,14 | + | ATCHS/CHS/TT4 (Chalcone synthase) |  |
| Isoflavonoid biosynthesis | Ta.11122.1.A1_at |  |  | 7,00 | - | ATCHS/CHS/TT4 (Chalcone synthase) |  |
| **Miscellaneous defense related genes** | |  |  |  |  |  |  |
| PPIase_Cyclophilin-type | Ta.9153.1.S1_at |  |  | 9,51 | + | peptidyl-prolyl cis-trans isomerase, putative |  |
|  | TaAffx.132123.1.A1_at |  |  | 2,93 | + | disease resistance-responsive protein-related |  |
|  | TaAffx.132123.1.A1_x_at |  |  | 2,77 | + | disease resistance-responsive protein-related |  |
|  | Ta.10273.1.S1_at |  |  | 2,07 | + | disease resistance-responsive family protein |  |
|  | Ta.5222.1.S1_at |  |  | 4,32 | + | disease resistance-responsive family protein |  |
|  | Ta.26046.1.A1_at |  |  | 2,05 | + | disease resistance protein RPM1, putative |  |
| **Transcription and signalling** | |  |  |  |  |  |  |
|  | Ta.25920.1.A1_at |  |  | 2,75 | + | myb-related protein |  |
|  | Ta.5490.1.S1_at |  |  | 2,30 | + | EF hand family protein, putative |  |
|  | Ta.5490.1.S1_x_at |  |  | 2,16 | + | EF hand family protein, putative |  |
|  | TaAffx.131308.1.S1_x_at |  |  | 2,04 | + | bZIP transcription factor domain containing protein |  |
|  | Ta.4862.1.A1_at |  |  | 2,05 | + | helix-loop-helix DNA-binding domain containing protein |  |
|  | TaAffx.24213.1.S1_at |  |  | 2,49 | + | TATA-box-binding protein 2 |  |
|  | TaAffx.10868.1.A1_at |  |  | 2,93 | + | MIKC-type MADS-box transcription factor WM24A |  |
|  | TaAffx.28919.1.S1_at |  |  | 2,06 | + | DHHC zinc finger domain containing protein |  |
|  | TaAffx.9161.1.S1_at |  |  | 2,10 | + | zinc finger, C3HC4 type domain containing protein |  |
|  | Ta.8671.1.S1_at |  |  | 2,68 | + | ZOS9-03 - C2H2 zinc finger protein |  |
|  | Ta.2912.1.S1_at |  |  | 2,18 | + | protein kinase domain containing protein |  |
|  | TaAffx.52428.1.S1_at |  |  | 3,31 | + | protein kinase domain containing protein |  |
|  | Ta.26118.1.S1_at |  |  | 3,38 | + | protein kinase family protein, putative |  |
|  | Ta.10236.2.S1_at |  |  | 2,96 | + | protein kinase domain, putative |  |
|  | Ta.18713.1.S1_s_at |  |  | 2,06 | + | receptor protein kinase, putative |  |
|  | Ta.7752.1.S1_at |  |  | 2,10 | - | RLK902 (receptor-like kinase 902) |  |
|  | Ta.6407.2.S1_at |  |  | 2,20 | - | protein kinase family protein |  |
| G protein-co+led receptors | Ta.10447.1.S1_at |  |  | 2,15 | - | Rho guanyl-nucleotide exchange factor |  |
| G protein-co+led receptors | Ta.10447.1.S1_a_at |  |  | 3,85 | - | Rho guanyl-nucleotide exchange factor |  |
| activated by G protein-co+led receptors | Ta.8726.1.S1_s_at |  |  | 2,37 | - | ATPLC2 (phospholipase 2); phospholipase C |  |
|  | TaAffx.40634.1.S1_x_at |  |  | 3,48 | - | phosphate-responsive protein, putative |  |
|  | Ta.11836.1.A1_x_at |  |  | 3,94 | - | phosphate-responsive protein |  |
|  | TaAffx.40634.1.S1_at |  |  | 4,04 | - | phosphate-responsive protein, putative |  |
| **Hormone metabolism** | |  |  |  |  |  |  |
| Auxine | TaAffx.105485.1.S1_at |  |  | 2,68 | + | auxin-induced protein 5NG4, putative |  |
| Auxine | Ta.9552.2.S1_at |  |  | 2,02 | + | auxin-induced protein 5NG4, putative |  |
| Auxine | Ta.4104.1.S1_at |  |  | 3,11 | + | auxin-responsive protein, putative |  |
| Auxine | Ta.4104.1.S1_s_at |  |  | 3,19 | + | auxin-responsive protein, putative |  |
| Salicylic acid | TaAffx.122522.1.S1_at |  |  | 2,15 | - | pentatricopeptide (PPR) repeat-containing protein |  |

Only significantly differentially expressed genes (absolute t-value >1.96 and ≥ 2 fold change) are shown in the table.
