# Supplementary material for: Jasmonate and ethylene dependent defence gene expression and suppression of fungal virulence factors: two essential mechanisms of Fusarium head blight resistance in wheat?
Source: BMC Genomics. 2012 Aug 2;13:369. doi: 10.1186/1471-2164-13-369 (PMC3533685; doi:10.1186/1471-2164-13-369)
Supplement: Additional file 4 — Table 4. Sequences of primers used for qPCR analysis of gene expression. Supplemental table showing sequences of primers that were used for the qPCR assays. Accession numbers of Expressed sequence tags (ESTs) and genes that were used to design primers are listed as well. All primers were designed using Primer3Plus software. [file 1471-2164-13-369-S4.doc]

| **Additional table 4: Sequences of primers used for qPCR analysis** | | | | |
| --- | --- | --- | --- | --- |
| **Probe Set** | **Description** | **Output sequence** | **Direction** | **Sequence (5' → 3')** |
| Ta.22614.1.S1_at | Serine protease inhibitor | CK196805 | F | tgacgggtgagttcaatgag |
|  |  |  | R | ccgtgcccatgcttatttat |
| Ta.8232.1.A1_at | TaPDR1 (pleiotropic drug resistance 1) | FJ185035 | F | gcattaagcacgatttcc |
|  |  |  | R | actttgacaacaaccgacag |
| Ta.2793.1.S1_at | TaMDR1 (MDR-like ABC transporter) | AB055077 | F | ctttcgctaccctgcaagac |
|  |  |  | R | gccgatcttccctcttatcc |
| Ta.23272.1.S1_at | TaUGT3 (UDP-glucosyltransferase protein) | FJ236328 | F | gttcgaggagcgtgtcaaag |
|  |  |  | R | acctgcacagatgccctcta |
| Ta.12887.1.S1_at | UDP-glucosyltransferase HvUGT13248 | BQ281752 | F | tcttgtgggtattccgcatt |
|  |  |  | R | ccttttgcatccacttcaca |
| Ta.8040.1.A1_at | Putative subtilisin-like serine proteinase | BQ161169 | F | cagcggaagcaacatatcatt |
|  |  |  | R | gggtacttccgtctgaccat |
| Ta.1207.1.S1_at | ZmOPR1 (12-oxo-phytodienoate reductase 1) | CA650490 | F | cggtggaggtaattgatgct |
|  |  |  | R | gtacatcccgagtgcatgtg |
| Ta.28553.1.S1_s_at | Ubiquitin | X56601 | F | ccctggaggtggagtcatctga |
|  |  |  | R | gcggccatcctcaagctgctta |
